# Formulation Feasibility of a Mechanically Compliant Stearate Organogel–Methylcellulose/Gelatin Bigel for Localized Neurotherapeutic Delivery

**DOI:** 10.3390/gels12070574

**Published:** 2026-06-29

**Authors:** Botle Matha Moswatsi, Gillian Dumsile Mahumane, Pradeep Kumar, Yahya Essop Choonara

**Affiliations:** Wits Advanced Drug Delivery Platform Research Unit, Department of Pharmacy and Pharmacology, School of Therapeutic Science, Faculty of Health Sciences, University of Witwatersrand, 7 York Road, Parktown, Johannesburg 2193, South Africa; 1405936@students.wits.ac.za (B.M.M.); gillian.mahumane@wits.ac.za (G.D.M.); pradeep.kumar@wits.ac.za (P.K.)

**Keywords:** bigel, traumatic brain injury, biphasic biomaterial, localized drug delivery, multiphasic drug release, cytocompatibility, organogel, hydrogel

## Abstract

Traumatic brain injury (TBI) presents a mechanically sensitive and pharmacologically complex environment in which therapeutic delivery remains challenging. Bigels may offer a formulation strategy for incorporating therapeutics with differing physicochemical properties while providing soft, viscoelastic matrices with properties that may be relevant to neural delivery applications. This study evaluated the in vitro formulation feasibility of a biphasic stearate organogel–methylcellulose/gelatin bigel as a mechanically compliant biphasic vehicle for localized delivery of neurotherapeutic agents. Bigels were fabricated by hot emulsification and genipin crosslinking to generate hydrogel-dominant dual-phase systems. Hydrogel:organogel formulations of 95:5 (BG1) and 85:15 (BG2) showed storage moduli of approximately 250 Pa and 200 Pa, respectively, and compressive Young’s moduli of 0.39 and 0.70 kPa, within reported ranges for soft brain tissue. Stress relaxation confirmed viscoelastic behaviour, while minimal oil leakage (<0.2%) indicated phase stability. BG1 showed 52% porosity, pore sizes of 1.8–22 µm, and approximately 14% weight gain. Drug release followed Weibull kinetics (R^2^ = 0.99–0.999), with nicotinamide showing faster release and N-acetylcysteine and TPGS showing more sustained release. Both unloaded and drug-loaded bigels maintained >70% PC12 cell viability. These findings support the formulation feasibility of biphasic bigels as mechanically compliant vehicles capable of accommodating therapeutics with differing physicochemical properties and exhibiting differential release behaviour. Further studies are required to evaluate degradation, tissue interactions, retention, and therapeutic performance in advanced in vitro and in vivo models.

## 1. Introduction

Traumatic brain injury (TBI) remains a leading cause of mortality and long-term neurological morbidity worldwide [1]. Its pathophysiology is inherently multimodal, characterized by a primary mechanical insult, followed by a complex cascade of secondary processes including neuroinflammation, oxidative stress, excitotoxicity, mitochondrial dysfunction, and progressive neuronal loss [2]. The temporal and spatial heterogeneity of these processes necessitates therapeutic strategies that address multiple biological pathways simultaneously. However, current pharmacological interventions remain largely inadequate in achieving this objective [3].

Systemic delivery approaches, such as oral or intravenous administration, are constrained by limited blood–brain barrier (BBB) permeability, rapid clearance, and subtherapeutic concentrations at the injury site [4,5]. Intranasal administration, while non-invasive, is associated with low drug absorption efficiency and inconsistent bioavailability [6,7]. Advanced interventions, including stem cell therapies and decompressive surgical strategies, have demonstrated partial benefits but remain limited by delivery constraints, survival challenges, and insufficient integration with host neural tissue [8,9]. Furthermore, large-scale clinical trials such as CRASH (corticosteroids) and PROTECT III (progesterone) have highlighted the translational challenges associated with single-agent pharmacotherapy in TBI [10,11]. Collectively, these findings underscore the need for localized, multimodal therapeutic platforms capable of overcoming both pharmacokinetic and microenvironmental constraints.

Beyond pharmacological limitations, the mechanical environment of neural tissue introduces an additional design constraint. Brain parenchyma exhibits a low elastic modulus (~0.1–1 kPa) and pronounced viscoelastic behaviour, characterized by time-dependent stress relaxation under physiological loading conditions [12,13]. Materials intended for intracranial application are generally designed to minimize mechanical mismatch with surrounding tissue in order to reduce the potential for strain-induced responses. Conventional hydrogel systems, although capable of mimicking extracellular matrix (ECM) hydration and softness, often exhibit rapid degradation, limited mechanical resilience, and poor encapsulation capacity for hydrophobic agents [14,15,16]. Hydrophilic matrices also exhibit unfavourable partitioning behaviour for lipophilic compounds (logP > 0), resulting in poor encapsulation efficiency or accelerated diffusion [17]. Organogels, conversely, provide lipid-rich matrices capable of encapsulating hydrophobic agents and sustaining their release, thereby improving local retention and pharmacokinetic stability [18,19,20]. However, organogels lack hydrophilic domains necessary for aqueous drug incorporation, protein interaction, and enzymatic biodegradation, and have not been widely explored in central nervous system (CNS) tissue engineering contexts [21]. The independent use of either hydrogels or organogels, therefore, presents intrinsic limitations in addressing the multimodal and mechanically sensitive requirements of TBI.

Bigels, biphasic systems integrating hydrogel and organogel networks, offer a potential solution by combining aqueous and lipid domains within a single structured matrix. Through phase ratio modulation, bigels can exhibit tunable rheological behaviour, shear-thinning properties, and adjustable storage modulus (G’), enabling control over injectability, retention, and mechanical compliance [20,21,22,23]. Importantly, the hydrogel-to-organogel ratio directly influences viscoelastic behaviour and network architecture, providing a design parameter for tuning mechanical properties toward reported neural tissue ranges while enabling co-delivery of hydrophilic and lipophilic agents.

Despite growing interest in bigels for dermal, transdermal, and localized drug delivery, their use as mechanically compliant vehicles for neurotherapeutic delivery remains insufficiently defined [21]. For neural applications, formulation design requires consideration of both physicochemical and biomechanical factors, including phase stability, accommodation of therapeutics with differing physicochemical properties, and mechanical behaviour relevant to reported soft neural tissue properties. This study, therefore, developed and characterized a stearate organogel–methylcellulose/gelatin bigel as a preliminary biphasic formulation platform for localized neurotherapeutic delivery. The methylcellulose–gelatin hydrogel phase was selected to provide a hydrated, biodegradable, and mechanically compliant matrix, while the stearate-based organogel phase was incorporated to facilitate the inclusion of lipophilic compounds. Protein-based hydrogel systems have been widely reported to form interconnected networks with improved structural integrity and biofunctionality compared with single-polymer systems [24]. This general behaviour has also been extended to polysaccharide–protein combinations, in which synergistic polymer interactions enhance mechanical performance and material robustness under physiological conditions [25]. In particular, hybrid hydrogel systems such as chitosan–gelatin and alginate–gelatin have demonstrated improved network cohesion and structural stability arising from complementary ionic and physical interactions between components, enabling multifunctional performance in responsive biomaterials [26,27]. Similarly, mechanically robust polysaccharide-based hydrogel systems have been shown to exhibit excellent resistance to structural collapse and swelling instability, contributing to long-term stability in aqueous environments [28].

Collectively, these systems highlight the advantage of combining polysaccharides with proteins to integrate mechanical integrity with biological functionality. Therefore, the combination of methylcellulose and gelatin was intended to leverage the structural support and thermoresponsive behaviour of the polysaccharide together with the biological relevance and flexibility of gelatin, promoting network formation and phase stability. This combination provides a rational basis for stable bigel formation and for accommodating therapeutics with differing physicochemical properties, thereby extending the functional versatility of the system toward potential biomedical applications. Furthermore, the dual-polymer system was selected to provide advantages over single-component hydrogels through enhanced phase stability, tunable mechanical behaviour, and accommodation of therapeutics with differing physicochemical properties. The study investigated physicochemical stability, structural architecture, thermal behaviour, mechanical properties, drug release kinetics, and in vitro cytocompatibility in order to assess the formulation feasibility of a mechanically compliant biphasic bigel system for localized neurotherapeutic delivery.

## 2. Results and Discussion

### 2.1. Formulation Development and Gelation Behaviour

#### 2.1.1. Preparation of Organogels

Soybean oil and stearic acid were used to prepare organogels (OGs). The OGs were initially translucent at 70 °C, transitioning to an opaque semi-solid state upon cooling to room temperature, reflecting stearic acid crystallization and the formation of a fibrillar network that immobilized the oil phase. The final OG exhibited a yellowish, firm texture, characteristic of the immobilization of soybean oil within the stearic acid network. The transition in opacity and texture suggests successful gelation, consistent with the formation of crystalline domains that surpass the percolation threshold, thereby immobilizing the continuous oil phase, as further confirmed by the inversion test which showed no gravitational flow (Figure S1), and Elastosens gelation time. This immobilization indicates the formation of a structured network capable of trapping the oil phase as shown by the minimal leaching (<0.2%) in B1 and BG2, highlighting that network density directly influences oil retention and may contribute to formulation stability in localized delivery applications. This was further supported by FTIR analysis, which shows the merged peaks of stearic acid and soybean oil, indicating the presence of both organogel components within the formulation. In addition, the characteristic peaks at 1180 cm^−1^ (C=O bending) and 1730 cm^−1^ (C=O stretching) confirm the presence of soybean oil and stearic acid within the bigel formulations, supporting the incorporation of soybean oil and stearic acid within the gel matrix [29].

#### 2.1.2. Preparation of Hydrogels

Gelatin and gelatin-methylcellulose hydrogels were prepared, with and without crosslinking using genipin (Figure S1). The yellowish colour observed in HG1 is attributed to gelatin, while the blue colour in HG2 indicates the presence of genipin. Genipin’s chromogenic reaction is well documented, with the characteristic blue coloration arising from the formation of genipin–amine crosslinked chromophores, typically associated with an absorbance band around 610 nm as reported in previous studies, and this colour change is indicative of successful crosslinking, reflecting covalent bond formation between genipin and primary amines, which has been reported to contribute to increased elastic modulus and reduced swelling in gelatin-based systems [30,31]. Crosslinking increased the network density of the hydrogels, thereby reducing polymer chain mobility and enhancing structural rigidity relative to uncrosslinked hydrogels, suggesting enhanced structural rigidity, due to the formation of stable covalent bonds between gelatin and genipin. The gelation of the hydrogels was also confirmed using the inverted tube method, where the formulations showed no flow under gravity, indicating the formation of stable hydrogels. The composite hydrogel (HG6), which included both gelatin and MC, had a pale-yellow colour and showed increased rigidity as confirmed by storage modulus (G’), suggesting network interactions between the gelatin and methylcellulose components.

Importantly, the incorporation of NAC and nicotinamide did not disrupt the gelation process as no visible phase separation or premature liquefaction was observed, suggesting that their incorporation did not adversely affect hydrogel formation under the conditions investigated.

#### 2.1.3. Preparation and Stability of Bigels

**Figure 1:**
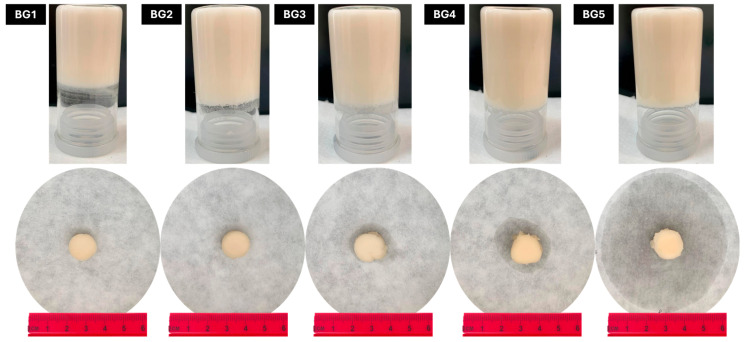
Photographs and corresponding leachate assessment following 24 h incubation (BG1 = 1.00 ± 0.06 cm, BG2 = 1.00 ± 0.10 cm, BG3 = 2.00 ± 0.20 cm, BG4 = 3.00 ± 0.11 cm, BG5 = 7.00 ± 0.26 cm). Pairwise comparisons demonstrated significant differences in leaching area between all formulations. The largest differences were observed between BG5 and the other samples (BG1–BG4, *p* < 0.001). Significant differences were also found among intermediate formulations (BG4 vs. BG1–BG3, BG3 vs. BG1–BG2, and BG4 vs. BG3; all *p* < 0.001). The smallest but still significant difference was between BG2 and BG1 (*p* = 0.024) (Figure S2).

Bigels were prepared by combining stearate organogels and gelatin-MC hydrogels in varying ratios of hydrogel to organogel (95%:5%, 85%:15%, 75%:25%, 65%:35%, and 50%:50% H:O in BG1–BG5, respectively. The hydrogel-to-organogel ratios were selected based on preliminary screening of physical appearance, phase homogeneity, and short-term stability using visual inspection and brightfield microscopy. Formulations showing phase separation or poor structural integrity were excluded, and the final ratios (95%:5% to 50%:50%) were selected to create a gradient from hydrogel-dominant to balanced bigels, enabling systematic evaluation of how increasing organogel content influences drug release kinetics and mechanical properties relevant to localized neurotherapeutic delivery applications. The stability of the bigels was assessed by leaching tests at 37 °C and 80% relative humidity as a preliminary formulation stability assessment. BG1 and BG2 exhibited minimal leaching (0.05 ± 0.29% and 0.20 ± 0.29% over 24 h, respectively) (Figure 1), consistent with improved phase stability. In contrast, leaching progressively increased with higher organogel fractions (BG3: 0.31 ± 0.29%, BG4: 1.00 ± 0.29%, BG5: 37.0 ± 0.29%), highlighting the critical role of composition in phase stability and oil retention (Table 1). This stability may be related to the network of stearic acid fibrils in the organogel phase, which effectively immobilized the oil and prevented leakage, a factor that may influence the retention and release behaviour of hydrophobic compounds. Formulations with higher organogel content appeared to exhibit reduced phase stability, likely due to insufficient hydrogel confinement and approaching the phase inversion threshold, which reduces the percolation of the continuous network and promotes oil release.

**Figure 2:**
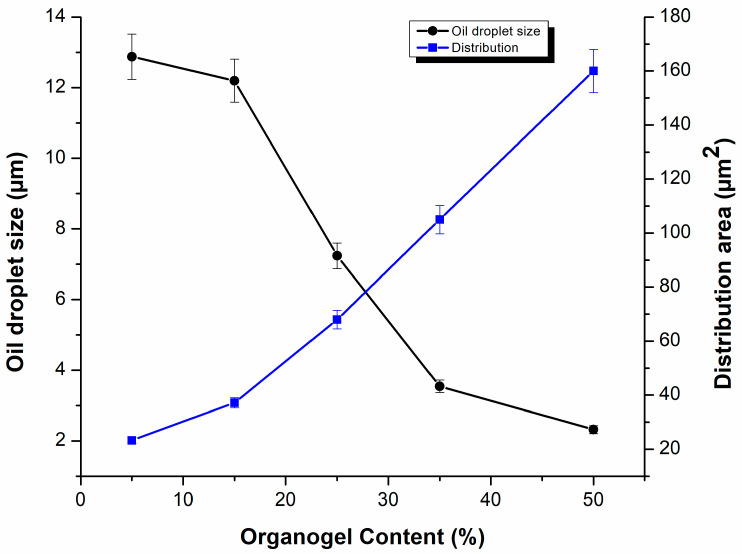
Droplet size and oil distribution area of bigels with varying hydrogel-to-organogel ratios. Increasing organogel content significantly decreased mean droplet size (from 12.87 µm in BG1 to 2.32 µm in BG5), while simultaneously broadening the distribution area (from 23.27 µm^2^ in BG1 to 159.99 µm^2^ in BG5). Error bars indicate standard deviation (SD).

The appearance of the bigels (milky white and creamy) is consistent with the dispersion of light at the interface between the hydrogel and organogel phases [32]. With the hydrogel forming the continuous phase and organogel droplets dispersed within it, droplet size decreased significantly with increasing organogel content (5.64–10.56 µm, Figure S3), suggesting that smaller, more uniform droplets may contribute to improved phase homogeneity and network organization. The soft, semi-solid texture of the bigels indicates successful co-gelation of the two phases, supported by rheological data (G’ > G”, frequency independence) and stress relaxation behaviour demonstrating the formation of a viscoelastic network, supporting the formation of a macroscopically homogeneous biphasic system. The presence of Tween^®^ 80 reduces interfacial tension between the organogel and hydrogel, leading to improved miscibility and phase stability (Figure 2). Tween^®^ 80 is known to improve emulsification and reduce interfacial tension in biphasic gels [33,34]. Although TPGS possesses surfactant properties and can form micelles above its reported critical micelle concentration (~0.02% *w*/*w*), it was incorporated in this study primarily as a model drug rather than as a structural component of the bigel. The concentration used (0.1% *w*/*w*) is relatively low in the context of the multi-component system and is unlikely to substantially affect phase behaviour dominated by the stearate organogel and methylcellulose/gelatin hydrogel matrices. Similar observations have been reported in other polymer- and lipid-based systems, where TPGS at low levels primarily acts as a solubilizer for hydrophobic compounds without significantly altering the bulk microstructure. Gelation was confirmed by the inverted tube method and further supported by rheological analysis, where frequency sweep data showed G’ > G” with storage moduli of ~200–250 Pa for BG1 and BG2, supporting their classification as viscoelastic, self-supporting gel systems. The bigels remained immobile under gravity, demonstrating that the formulations maintained structural integrity under the conditions evaluated (Figure 1).

#### 2.1.4. Selection of Optimal Bigel for Further Analysis

BG1 and BG2 were selected based on minimal leaching (<0.2%), uniform consistency, and rheological properties (G’ = 250 ± 1.26 and 200 ± 0.64 Pa) for further analysis, including swelling studies, drug release, and mechanical testing. This decision was supported by the observation of minimal oil leaching, which is a key indicator of bigel stability in formulations intended for controlled drug release. Additionally, the smooth texture of the two formulations supported their selection for further physicochemical, mechanical, and drug-release characterization. The addition of nicotinamide, NAC, and TPGS to BG2 did not affect the structural integrity or gelation behaviour, as confirmed by FTIR, where distinct drug-associated peaks were not observed and appeared to be masked by the gelatin and stearic acid signals within the bigels. This was also observed in XRD where reduced peak intensity and decreased crystallinity suggested partial amorphization of the composite system, highlighting the potential of BG2 as a biphasic delivery platform capable of accommodating both hydrophilic and hydrophobic compounds. The dual-phase may facilitate differential release behaviour of compounds with differing physicochemical properties, warranting further investigation in neurotherapeutic delivery applications.

### 2.2. Microscopy Analysis

#### 2.2.1. Bright-Field Microscopy

The microstructure of the organogels (OG), hydrogels (HGs), and bigels (BGs) was examined using bright-field microscopy to evaluate the phase distribution between the hydrogel and organogel components (Figure S3). The composition and coding of the different formulations are outlined in Table 2, detailing varying ratios of organogel to hydrogel.

Brightfield microscopy images (Figure S3) of the bigel formulations (BG1 to BG5) reveal distinct microstructure and phase distribution differences, influenced by the changing hydrogel–organogel ratio. Small, dispersed circular droplets were observed, suggesting a well-dispersed organogel phase within the hydrogel matrix, as seen in prior studies [29]. All bigels showed aggregates of oil droplets, which may reflect localized droplet clustering within the biphasic matrix. In the organogel phase, stearic acid forms a crystalline fibrillar network, which may contribute to droplet clustering at higher organogel fractions. BG1 (95%/5% hydrogel-to-organogel) appears continuous, with minimal visible oil droplets, indicating a continuous hydrogel phase with isolated organogel regions. As the organogel content increases, BG2 (85%/15%) presents more prominent organogel clusters, with increased aggregation of oil droplets within the hydrogel matrix, demonstrating a more dispersed oil phase. The BG3 (75%/25%) and BG4 (65%/35%) display more dispersed structures with possible phase separation, suggesting reduced phase homogeneity relative to BG1 and BG2. In BG5 (50%/50%), the hydrogel and organogel phases appear less integrated, resulting in a heterogeneous system.

Quantitative analysis of the droplet size and distribution supports these observations (Figure 2). There was a significant decrease in droplet size with increasing organogel content, ranging from 5.64 µm (BG1 vs. BG3, *p* < 0.001) to 10.56 µm (BG1 vs. BG5, *p* < 0.001). These differences were observed between the hydrogel-rich (BG1/BG2) and organogel-rich (BG4/BG5) formulations. In contrast, no significant difference was found between BG1 and BG2 (*p* = 0.202) and between BG4 and BG5 (*p* = 0.272). indicating that the size stabilizes at both ends of the compositional spectrum. On the other hand, as expected, oil distribution increased with an increase in organogel content, and the biggest difference was observed between BG1 and BG5 (130.32 µm^2^, *p* < 0.001), which may reflect increasing aggregation and spatial distribution of hydrophobic domains as organogel content increased. There was a progressive and significant increase in the distribution between BG2–BG3 (*p* = 0.042) and BG3–BG5 (*p* = 0.026), highlighting a gradual transition toward heterogeneity. Only BG1 versus BG2 (*p* = 0.233) demonstrated a non-significant difference.

The bicontinuous phase structure observed in BG1 and BG2 may contribute to the higher viscoelastic properties observed during rheological analysis. These samples showed higher storage modulus (G’) dominance over loss modulus (G”), indicating predominantly elastic gel behaviour over the frequency range investigated. The methylcellulose–gelatin hydrogel phase likely contributed to the formation of an interconnected network that stabilized dispersed organogel droplets and maintained structural integrity. These observations are consistent with previously reported gelatin/alginate hydrogel systems where elastic recovery is improved due to dual-network stabilization through cooperative hydrogen bonding and physical entanglement [35]. Individually, methylcellulose and gelatin are limited, as methylcellulose lacks strong cohesive bio-polymeric reinforcement at physiological conditions, whereas gelatin alone exhibits weak mechanical stability and thermal sensitivity near physiological temperature. Their combination therefore enables a complementary dual-network architecture in which methylcellulose provides structural percolation and gelatin reinforces interchain cohesion, resulting in improved droplet immobilization and resistance to phase separation compared to single-polymer systems.

In contrast, BG3 displayed a more dispersed phase characterized by droplet-like domains indicative of phase separation, which corresponded with lower viscoelastic properties observed in rheological analysis and increased oil leakage relative to BG1 and BG2 BG4 and BG5 exhibited a more emulsion-like morphology with a moderate increase in G’ and G” at higher frequencies, suggesting intermediate gel strength and structured fluid-like behaviour. The observed differences highlight the influence of microstructural organization on the physicochemical and rheological behaviour of bigel formulation.

As the organogel content increased from BG1 to BG5, the mean oil droplet size decreased while the overall distribution area increased, indicating changes in phase organization and spatial distribution within the biphasic matrix. These observations suggest progressive changes in microstructural organization with increasing organogel content, which is critical for achieving balanced hydrophilic-hydrophobic interactions in bigels. Such a biphasic structure may facilitate differential partitioning of hydrophilic and hydrophobic compounds between the hydrogel and organogel domains, supporting further investigation of these systems as localized drug-delivery platforms.

#### 2.2.2. Scanning Electron Microscope (SEM)

**Figure 3:**
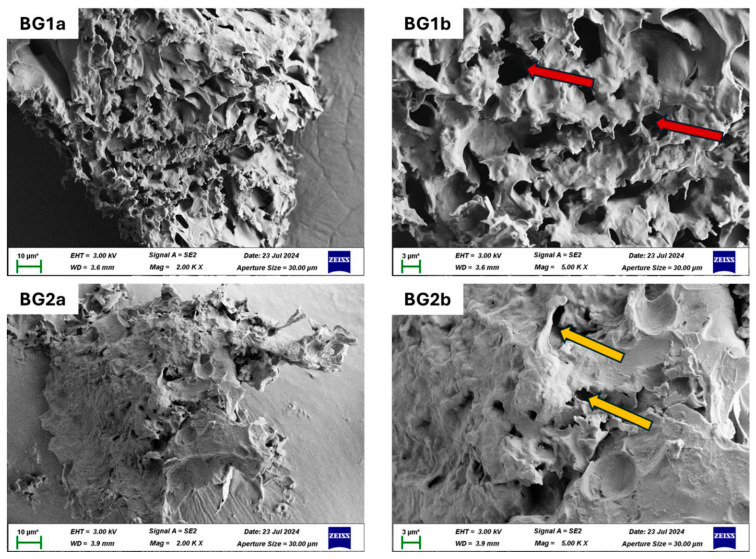
Scanning Electron Microscopy (SEM) images of BG1 and BG2 (a: 2000×, b: 5000×), highlighting surface morphology and apparent porous microstructure of the dehydrated bigel systems. Pores are indicated with arrows. Colours are only used as a visual aid to distinguish and highlight pore regions. * Samples were analyzed at an accelerating voltage of 10 kV under high vacuum conditions.

To study the surface morphology and microstructural characteristics of the bigels, particularly focusing on BG1 and BG2, the bigels were visualized under SEM, as shown in Figure 3. Quantitative analysis of the SEM images was also performed using ImageJ software version 1.54t (NIH, Bethesda, MD, USA) to determine pore diameter and percentage porosity. These SEM images give a detailed view of the internal network and porosity of the bigel; however, it should be noted that the observed structures reflect the dehydrated scaffold architecture rather than the in situ hydrated structure.

BG1 (95%:5% hydrogel-to-organogel) exhibits a porous, interconnected network (52% porosity), whereas BG2 (85%:15%) shows a denser, tightly interconnected structure with relatively fewer visible pores (10.2% porosity). Increasing the organogel fraction reduces apparent porosity because hydrophobic organogel domains lower the overall water content of the formulation. During freeze-drying, less water is available to form ice crystals, which normally act as templates for pore formation. As a result, the network contains fewer or smaller pores, and the apparent porosity of the bigel decreases with higher organogel content. The larger pores and more open network observed in BG1 are consistent with a structure that may facilitate fluid penetration and molecular transport within the matrix. In contrast, BG2 exhibited a more compact structure with lower apparent porosity, which may influence molecular diffusion and release behaviour relative to BG1. These observations may reflect differences in transport behaviour associated with variations in pore structure and network density (the Weibull release constants for NAC, nicotinamide, and TPGS were k = 0.43, 0.42, and 0.41, respectively), where increased network density and reduced pore connectivity may reduce molecular mobility within the matrix and contribute to the more sustained release profiles observed.

Quantitative analysis of pore size and porosity confirmed these observations. The BG1 exhibited an average pore size ranging from 1.8 to 22 µm with a porosity of 52%, while BG2 showed smaller pores ranging from 0.023 to 10.8 µm with 10.5% porosity. Studies indicate that an adult human stem cell is approximately 20 µm, and pluripotent stem cells are typically 3 to 5 µm, whereas large mesenchymal stem cells range from 30 to 50 µm. Therefore, the pore size distribution observed in BG1 may provide a microstructure that could be explored in future studies investigating cellular interaction with the matrix. BG2’s smaller pores may contribute to reduced diffusional pathways and altered release, consistent with the Weibull release constants obtained for NAC (k = 0.43), nicotinamide (k = 0.42), and TPGS (k = 0.41). The observed differences between BG1 and BG2 demonstrate the influence of hydrogel:organogel ratio on microstructural organization and apparent porosity.

### 2.3. Molecular Characterization

**Figure 4:**
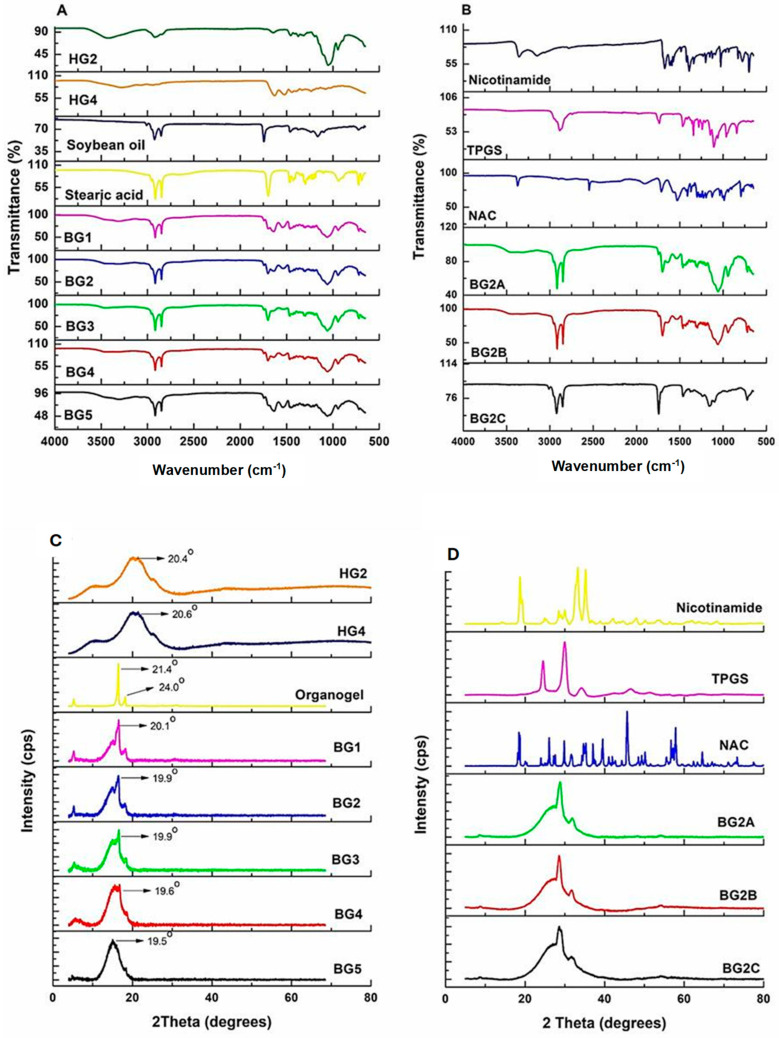
The FTIR spectra of (**A**) formulation components and bigels and (**B**) pure drugs and drug-loaded bigels, and pure drugs XRD profiles of (**C**) the bigels, organogels, and hydrogels, and (**D**) pure drugs, and drug-loaded bigel. The diffraction spectra were baseline-corrected and smoothed before analysis.

The FTIR spectra presented in Figure 4 provide insights into the chemical interactions and molecular characteristics of the hydrogels, bigels (Figure 4A), and drug-loaded bigels (Figure 4B). The broad peak at ~3234 cm^−1^ in HG4 is attributed to the stretching of N-H and O-H bonds, suggesting potential hydrogen bonding within the hydrogel network. This region is also relevant for crosslinking, as genipin reacts primarily with the ε-amino groups of lysine residues in gelatin to form covalent bonds. Such reactions can lead to changes in hydrogen bonding interactions, often observed as peak broadening or slight shifts within the 3200–3400 cm^−1^ range in FTIR spectra. These spectral modifications are consistent with structural rearrangements that may occur following genipin-mediated crosslinking. The amide I stretching and amine II (N-H) bending peaks at 1655 cm^−1^ and 1544 cm^−1^, respectively, corresponding to the characteristic protein backbone vibrations from gelatin [36]. These peaks were observed in the bigels (BG1–BG5), indicating the presence of gelatin-derived functional groups within the bigel formulations, and showed slight broadening and changes in position and intensity. These changes in the Amide I/II bands are consistent with altered hydrogen-bonding environments, electrostatic interactions, or network formation arising from formulation components (e.g., genipin crosslinking and interactions with oil/surfactant phases). Genipin reaction with primary amines (lysine residues) and subsequent covalent crosslink formation can increase intermolecular hydrogen bonding and structural heterogeneity, which typically show broadening and shift in the 3200–3400 and 1600–1500 cm^−1^ regions. Additionally, the absorption band at 1063 cm^−1^ corresponds to the C-O-C stretching of MC (HG2) in the hydrogels, suggesting the successful incorporation of MC into the bigel matrix.

All bigels showed peaks at 2933 cm^−1^ and 2871 cm^−1^, which are associated with the C-H asymmetric and symmetric stretching of the stearic acid and soybean oil present in the organogels [37,38]. The merged peaks of stearic acid and soybean oil reflect the saturated C18 fatty acid chains of stearic acid, indicating the successful integration of the organogel components [39]. The presence of characteristic peaks at 1180 cm^−1^ and 1730 cm^−1^ (representing C=O bending and stretching, respectively) further supports the presence of soybean oil and stearic acid in the bigel formulations. The characteristic peaks of stearic acid are ~1180 cm^−1^, ~1730 cm^−1^, ~2871 cm^−1^, and ~2930 cm^−1^ [40]. In the drug-loaded bigels, no distinct FTIR peaks corresponding to NAC, TPGS, or nicotinamide were observed. In addition to the relatively low concentrations of the drugs, this absence may be attributed to the low drug loading and overlap of drug-associated bands with stronger polymer and lipid signals within the matrix which may limit the detectability of drug-specific peaks within the composite spectrum [41]. These findings are consistent with incorporation of the drugs into the formulations without the appearance of new detectable functional groups. Further analyses using complementary techniques such as NMR could more conclusively confirm the molecular dispersion of the drugs within the matrix.

X-ray diffractograms of the (Figure 4C) bigels, hydrogels, and organogel, and (Figure 4B) pure drugs and drug-loaded BG2 are shown in Figure 4. The XRD patterns exhibit diffraction peaks at ~2θ = 6°, ~20°, and ~24°. The sharp and intense peak at ~2θ = 20° is characteristic of the presence of β-polymorph (orthorhombic) of stearic acid in the organogel [29,42,43]. This peak becomes broader and less intense in BG1 to BG5, suggesting increased structural disorder and reduced crystallinity within the composite matrices. This observation is further supported by the crystallinity index (C_i_) (Table S1), which shows a progressive decrease from 0.73 for OG (pure organogel) to 0.37, 0.29, 0.25, 0.24, and 0.20 for BG1 to BG5, respectively. The progressive reduction in Ci with increasing organogel fraction may be attributed to phase confinement effects, where the dispersed hydrogel domains within the organogel matrix restrict lamellar packing and crystal growth of the stearic acid, leading to a more disordered, partially amorphous structure. The reduction in Ci quantitatively supports reduced crystallinity and an increase in amorphous character with increasing organogel content. Stearic acid typically exhibits a characteristic crystalline peak, often in the range 2θ = 20–24°, hence, the peaks at 2θ = 20° and 24° are related to stearic acid [29]. The shift in peak positions and corresponding d-spacings for stearic acid with ~10% increase in interplanar spacing upon incorporation of organogels into the bigel matrix may reflect polymer-lipid interfacial interactions, confinement effects, or partial polymorphic transitions. This was further evaluated by calculating the d-spacings of the bigels with reference to that of stearic acid using Equation (1), Bragg’s law:

(1) nλ = 2dsinθ

where *n* is an integer (usually 1); *λ* is the CuKα wavelength, i.e., 1.54 Å; *d* is the d-spacing (interplanar spacing); *θ* is the Bragg’s angle (the angle at which the diffraction occurs) [44]. An increase in d-spacing indicates greater separation between diffracting planes and may reflect structural changes such as altered lamellar packing, interfacial interactions with hydrogel components, or reduced crystallinity within the bigel matrix. The d-spacings and C_i_ of the bigels, hydrogels, and stearic acid have been tabulated in Table S1. Overall, it was observed that the bigels exhibit a partially crystalline structure, with BG1 more crystalline than BG2, as confirmed by the C_i_ values, where BG1 has a higher C_i_ than BG2.

### 2.4. Thermal Analysis 

**Figure 5:**
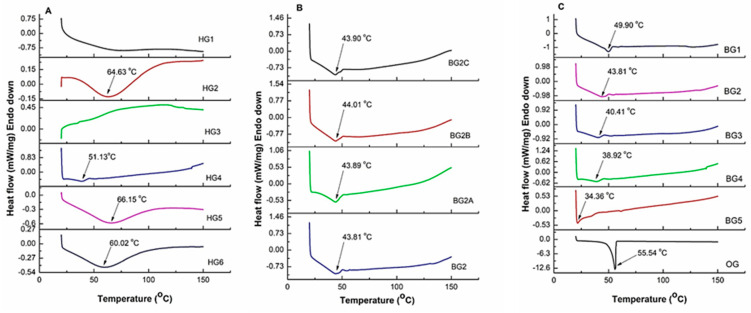
DSC thermograms of the formulations (**A**): hydrogels, (**B**): drug-loaded bigels, and (**C**): organogel and pure bigels.

Thermal analysis of the bigels and hydrogels was conducted using differential scanning calorimetry, as depicted in Figure 5. The freeze-dried hydrogels (Figure 5A) display distinct thermal transitions. Samples HG2 and HG4 show endothermic peaks at 64.63 and 51.13 °C, respectively, indicating a thermal transition associated with polymer-chain rearrangement and/or loss of bound water within the freeze-dried hydrogel network. These peaks may reflect thermally induced molecular rearrangements, influenced by the degree of crosslinking with genipin, and may be attributed to gelatin helix-coil transitions and/or the loss of bound water typically observed in lyophilized hydrogels. HG5 and HG6 exhibit slightly lower transition temperatures with HG6 showing a peak at 60.02 °C, potentially due to the presence of gelatin in the formulation, which may influence network organization and thermal behaviour.

For the bigels, BG1 to BG5 (Figure 5C), endothermic peaks ranging from ~34.36–49.90 °C were observed, indicating the solid–liquid transition temperatures of the bigels. Notably, these temperatures are lower than the peak observed for the stearate–organogel (~55.54 °C), suggesting that the incorporation of hydrogels into the organogel matrix significantly altered the thermal behaviour of the organogels [29,45]. This shift in thermal behaviour suggests that the hydrogel matrix modulates the thermal properties of the organogel, creating a composite material with altered thermal transition behaviour. On the other hand, the thermal behaviour of the bigel formulations, characterized by endothermic peaks ranging from ~34.36 to 49.90 °C, suggests phase transitions that may influence drug release kinetics. Given that physiological temperature (~37 °C) falls within this range, partial softening of the hydrogel–organogel network could enhance drug diffusion. The initial burst release phase, observed in drug release may be associated with surface-associated or weakly entrapped drug molecules, which may be further accelerated if the bigel matrix undergoes thermal softening at this temperature. Nicotinamide, being highly hydrophilic, exhibits the fastest release, which may be facilitated by increased hydrogel swelling and reduced matrix rigidity near the transition temperature. Conversely, NAC and TPGS, which may exhibit greater association with the organogel-containing domains, demonstrate a more controlled release profile, particularly in BG2, where the higher organogel content may contribute to retention of structural organization during heating. The presence of a hydrophobic organogel barrier in BG2 contributes to sustained drug release, mitigating the effects of temperature-induced softening and contributing to the more sustained release profiles observed experimentally. These findings suggest that thermal transitions may contribute to the release behaviour observed in the formulations.

BG1, with the highest hydrogel content, shows the highest thermal transition temperature, while BG5 (with the highest organogel content) shows the lowest thermal transition temperature, as increasing organogel fraction dilutes the crosslinked hydrogel network and introduces lipid domains that reduce the overall thermal transition of the composite. The bigels peaks are depreciating from BG1 to BG4, while the BG5 peak is not visible, suggesting reduced crystallinity within the composite systems. This pattern correlates with the trend observed in XRD profiles of the bigels in Figure 4 above. This trend indicates that increasing the organogel content influences the thermal transition behaviour of the bigels.

### 2.5. Leaching Studies

The leaching studies revealed important insights into the stability of the bigels. The results are presented in Figure 1 and Figure S4, and Table 1, showing distinct differences between the formulations.

The leaching data demonstrated that formulations with hydrogel content, specifically BG1 and BG2, exhibited minimal to no leaching (<0.2%), which is within the acceptable leaching limits [46]. In contrast, BG5, with the highest organogel content, displayed significant percentage oil leaching (35.8 ± 2.47 × 10^−3^%) after 24 h, indicating that such formulations exhibited reduced phase stability and greater oil leakage under the conditions investigated. The incorporation of the drugs into BG2 does not significantly change the percentage oil leaching from the bigels. It should also be noted that BG2B, with 20 mg of added compounds, shows the lowest leaching percentage (0.12 ± 4.91 × 10^−2^%), suggesting that this formulation exhibited the lowest oil leakage among the drug-loaded formulations investigated.

In contrast, BG2A and BG2C, with 10 mg and 50 mg of drugs, respectively, show higher percentage oil leaching (0.18% and 0.25 ± 1.48 × 10^−1^%), suggesting that oil leakage varied among the drug-loaded formulations; however, the underlying mechanisms remain unclear and require further investigation. However, further exploration via rheological and interfacial tension measurements is required. All formulation comparisons showed significant differences (*p* < 0.001). Specifically, BG5 showed significantly higher leaching compared with BG1, BG2, BG3, and BG4 (*p* < 0.001), while BG4, BG3, and BG2 also differed significantly from BG1 (*p* < 0.001). These findings indicate an association between increasing organogel content and increased oil leakage under the conditions investigated. The low oil leakage observed for BG1 and BG2 demonstrates improved phase stability relative to formulations containing higher organogel fractions. This improved phase stability in BG1 and BG2 can be attributed to the dominance of the methylcellulose–gelatin hydrogel network, which forms a physically entangled, interpenetrating polymer matrix where gelatin contributes cohesive protein-based junction zones through triple-helix formation, while methylcellulose provides a thermally reinforced continuous phase, together producing a barrier to oil diffusion and phase separation. This trend is consistent with recent reports on gelatin-polysaccharide hybrid hydrogels, where enhanced phase stability is achieved through interpenetrating polymer networks that restrict dispersed phase mobility and suppress oil/water phase separation under storage conditions. For example, Mori and colleagues demonstrated that gelatin-alginate composite hydrogels form a homogeneous hydrogel without cracks or phase separation, suggesting improved structural retention and reduced phase separation, highlighting the role of dual-polymer architectures in stabilizing multiphase systems [47].

However, further studies in phosphate-buffered saline, simulated cerebrospinal fluid, and biologically relevant environments are required to evaluate longer-term formulation stability and performance.

### 2.6. Swelling and Weight Loss Studies

Swelling studies provide important insights into water uptake, matrix hydration, and structural stability, all of which may influence formulation performance and drug-release behaviour. The brain’s composition comprises water, lipids, proteins, and other organic molecules, with approximately 75–80% of its mass consisting of water, which contributes significantly to its mechanical and biochemical properties [48].

**Figure 6:**
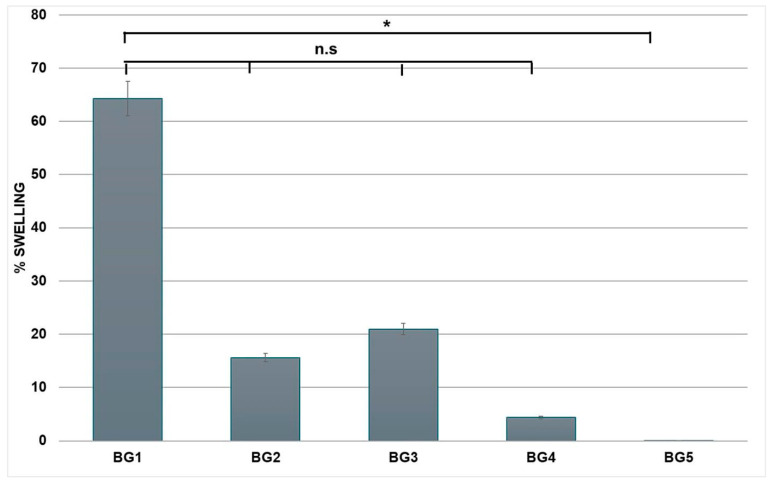
Initial swelling percentage of bigel formulations (BG1–BG5) measured at 0.5 h at 37 °C, swelling was assessed in phosphate-buffered saline (PBS). Data are presented as mean ± standard deviation (SD). Gradual deswelling and mass loss occurring after this time are reported in Table S2. The * indicates a significant difference (*p* < 0.05), while n.s indicates no significance.

The swelling behaviour of bigel formulations (BG1, BG2, BG3, BG4, and BG5) was assessed at 37 °C in PBS (Figure 6) to evaluate the influence of hydrogel:organogel ratio on hydration behaviour and matrix stability. At the early time point (0.5 h), BG1 exhibited the highest swelling (~60%), followed by BG2 and BG3 with moderate swelling capacities, while BG4 and BG5 showed minimal hydration (Figure 6). This initial phase reflects rapid water uptake driven by the gelatin/methylcellulose hydrophilic polymer network. Compared to the other formulations, BG5 showed a significant difference (*p* < 0.05 for all comparisons with BG1–BG4), indicating that higher organogel content markedly suppresses water uptake. BG2, BG3, and BG4 initially showed moderate swelling, which decreased over time, indicating reduced water retention relative to BG1. Differences among these formulations were not statistically significant (*p* > 0.05 for all pairwise comparisons).

Beyond 1 h, a progressive decrease in swelling was observed, with most formulations showing mass loss (Table S2). This transition from swelling to mass loss suggests that after reaching peak hydration, the gels underwent de-swelling and matrix erosion. However, dedicated hydrolytic and enzymatic degradation studies are required to confirm biodegradation behaviour. Such behaviour may influence the residence time and structural persistence of the formulation. The weight loss observed from 2 h onward may be associated with gelatin dissolution and hydrolytic erosion of the hydrated matrix at physiological temperature, consistent with FTIR evidence of polymer–water interaction and network relaxation. Gelatin exhibits characteristic peaks corresponding to amide I (~1650 cm^−1^), amide II (~1550 cm^−1^), and amide III (~1230 cm^−1^) bands, primarily related to protein secondary structures, as shown in the FTIR study above (Figure 5 HG2). These spectral changes may reflect alterations in polymer-chain interactions following hydration and drying. This is evident in BG2 to BG5, with a slight shift in the amide I peak, while the amide II peak disappears entirely, consistent with structural changes within the gelatin-containing network (Figure S4).

It is also important to note that the hydrogel absorbs water, whereas the organogel acts as a more hydrophobic phase, and the interaction between these two phases may limit the hydrogel’s ability to swell fully. The lipid peaks of the organogel, observed in the FTIR spectrum above (Figure 5) (2800–3000 cm^−1^), become more intense moving from BG1 to BG5 (Figure S4), indicating organogel dominance. This feature, along with the reduced O-H and N-H water-associated peaks (3300 cm^−1^), which may contribute to the reduced swelling observed at higher organogel contents. BG1’s strong swelling profile across different time points suggests a formulation with high water affinity. Conversely, BG5’s low swelling reflects a more hydrophobic structure, as it contains a greater amount of organogel compared to the other bigels. Overall, BG1 exhibited the highest swelling capacity among the formulations investigated, reflecting greater water uptake and matrix hydration relative to the other bigels. In addition, future work will evaluate hydrolytic and enzymatic degradation under physiologically relevant conditions to better characterize long-term formulation behaviour.

### 2.7. Mechanical Analysis

**Figure 7:**
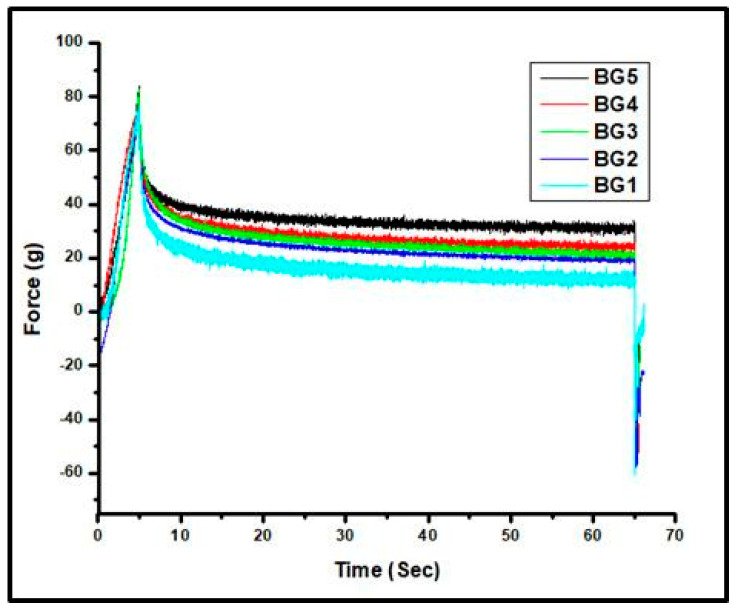
Stress relaxation curve of the bigels.

Figure 7 displays the stress relaxation curves of the bigels, demonstrating their mechanical response to a constant strain over time. The stress relaxation profiles of the bigels reveal important insights into the elasticity and viscoelastic response of the gel network, both of which are important considerations in the design of biomaterials intended for soft-tissue applications. All bigels exhibited a sharp initial increase in stress, corresponding to the immediate elastic response under the applied strain.

BG1 exhibited the fastest stress relaxation, with a rapid decrease over time. This indicates that BG1 loses its resistance to deformation more quickly than the other formulations, resulting in lower final stress values. Although BG1 displays rapid stress relaxation, its relatively high G’ indicates a structurally strong but viscoelastic network, potentially relevant for soft-tissue applications requiring viscoelastic stress dissipation. The rheological analysis of BG1 revealed a higher storage modulus (G’) compared to other bigels, indicating greater elastic strength and structural integrity. The higher G’ of BG1 is consistent with a continuous hydrogel phase contributing predominantly to stress bearing. This continuous network also translates into improved mechanical strength of the overall bigel system, where the interpenetrating dual system hydrogel matrix enhances resistance to deformation and helps maintain structural integrity under applied stress conditions. The mechanical stiffness through primary genipin–amine covalent crosslinking also contributes to the observed viscoelastic behaviour. Additionally, hydrogen bonding between methylcellulose and gelatin, as well as hydrophobic interactions within the organogel phase (soybean oil–stearic acid network), may contribute to the mechanical reinforcement but do not alter genipin crosslinking. Importantly, this mechanically reinforced network contributes directly to reduced phase instability, as the strengthened hydrogel framework limits oil droplet mobility and suppresses migration of the organogel phase, thereby mitigating leakage as seen in Figure 1. This mechanism was also observed in emulsions containing gelatin-sodium alginate double network hydrogels where the presence of a reinforced gel network enhances emulsion stability by suppressing droplet coalescence and preventing phase separation through steric and interfacial stabilization [49].

However, despite its high G’, BG1 also exhibits a rapid stress relaxation profile, signifying its ability to efficiently dissipate applied stress over time. This behaviour may be due to a high-water content and the dynamic nature of hydrogen bonding and weaker van der Waals forces within the hydrogel and organogel matrix, enabling reversible chain orientation. Despite strong covalent crosslinks, BG1 relaxes faster due to a lower effective crosslink density, higher solvent content, and dominance of reversible physical interactions, which together allow rapid polymer chain rearrangement and stress dissipation. In contrast, BG5, with the highest organogel content, showed the slowest relaxation and the highest final force value. The slower relaxation implies a more rigid, elastic gel network, where the organogel phase contributes significantly to the mechanical stability of bigel [45]. The higher final force values in BG5 indicate that the gel retains more of its resistance over time, highlighting its more elastic and resilient network.

However, from a formulation-design perspective, BG1 demonstrated mechanical properties that were closer to reported soft brain-tissue ranges than formulations containing higher organogel fractions. Brain tissue has relatively soft and elastic properties; materials intended for implantation are commonly designed to have similar mechanical properties to avoid adverse reactions or mechanical mismatch with the brain’s natural environment [50,51]. The quick relaxation and lower final force values observed in BG1 indicate a modulus within the reported range for brain tissue and demonstrate viscoelastic stress dissipation over the 60 s testing window.

In addition to relaxation profiles, the stress–strain characteristics of the gels further highlight their mechanical suitability for brain tissue applications (Figure S6). BG1 (95/5% hydrogel/organogel) exhibited a Young’s modulus of 393.01 Pa (0.39 kPa), while BG2 (85/15%) showed a modulus of 703.35 Pa (0.70 kPa). Both values fall within the 0.1–1 kPa range typically reported for native brain tissue, indicating mechanical relevance. Healthy brain tissue exhibits an elastic modulus typically ranging from 0.1 to 1 kPa, depending on the strain rate and measurement technique, while its relaxation modulus demonstrates significant stress dissipation, with white matter relaxing by over 70% within 300 s [52,53,54]. Injured brain tissue often exhibits altered mechanical properties such as changes in stiffness and viscoelastic behaviour. The study’s measurements over 60 s capture the initial viscoelastic response but do not fully reflect longer-term tissue relaxation. The observed increase in modulus from BG1 to BG2 suggests a clear dependence on formulation composition. This trend may be attributed to the increasing contribution of the organogel phase, which likely enhances structural rigidity through hydrophobic domain formation within the methylcellulose–gelatin network. Kim and colleagues have demonstrated that the double-network hydrogels exhibit substantially improved mechanical properties, including tensile strength and elastic modulus, compared to single-network gelatin hydrogels, which further reiterates the importance of the methylcellulose–gelatin hydrogel system used int his study [55]. In contrast, hydrogel-rich systems are dominated by the hydrated polymer matrix, resulting in lower stiffness and greater compliance and this indicates the effect on hydrogel ratios on mechanical tuning. BG1 and BG2 exhibited compressive modulus and storage modulus values within reported brain tissue ranges and showed viscoelastic stress dissipation under short-term compressive loading, supporting feasibility for further neurotrauma-relevant evaluation.

**Figure 8:**
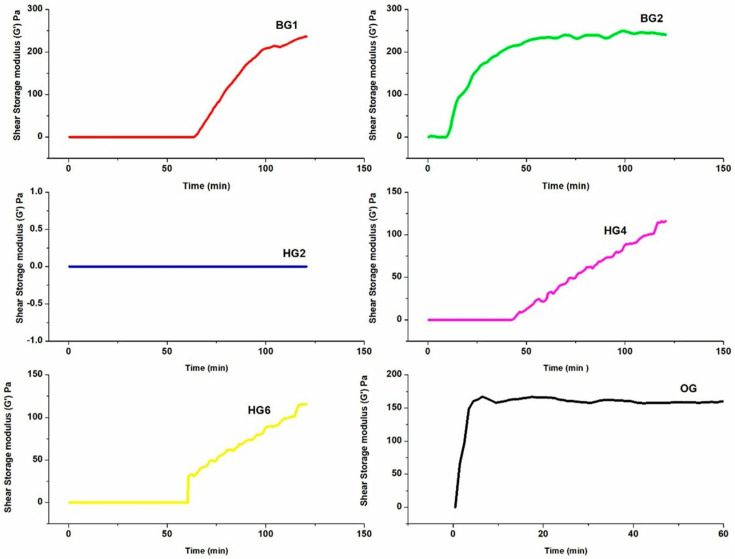
Gelation kinetics of the bigels, hydrogels, and organogel.

Figure 8 shows the gelation kinetics of the bigels (BG1 and BG2) and hydrogels (HG2, HG4, and HG6) as measured by the shear storage modulus (G’), which reflects the gel network strength and elasticity over time. The storage modulus (G’) is an important rheological parameter that indicates the solid-like behaviour of the gels and their ability to resist deformation.

BG1 shows the highest storage modulus (G’), reaching 250 ± 1.26 Pa, indicating that it formed the highest storage modulus among the formulations investigated. The rapid increase in G’ around 50 min, followed by a plateau around 110 min, suggests that the gel structure becomes substantially complete at this point. On the other hand, BG2 demonstrates a more gradual increase in G’, reaching its plateau at ~200 ± 0.64 Pa after about ~70 min. The earlier onset of gelation in BG2, occurring at about 10 min, is attributed to the differences in phase composition affecting network assembly, which likely enhances the crosslinking dynamics between the organogel and hydrogel phases. Faster gelation in BG2 may also be driven by quicker phase separation and diffusion of genipin, a crosslinker known to promote network formation [56]. Among the hydrogels, HG6 shows the highest G’ value, reaching ~125 ± 0.66 Pa after 120 min. This reflects the thermoresponsive gelation of methylcellulose, which transitions from a soluble state to a gel upon heating above its gelation temperature, typically around 40–60 °C [56,57]. The presence of MC in HG6 enhances the mechanical strength of the hydrogel network. HG4 (a crosslinked gelatin formulation) also shows a gradual increase in G’, reaching ~115 ± 0.69 Pa, demonstrating its ability to form a stable gel at 37 °C. Although MC typically gels at 40–60 °C, incorporation with gelatin may allow partial network formation at 37 °C, contributing to measured G’. HG2 (gelatin only) shows no gelation at 37 °C, as gelatin typically gels at temperatures below 30 °C due to its hydrogen bonding interactions. The absence of gelation in HG2 highlights the temperature sensitivity of gelatin, which does not form a gel network at physiological temperatures. Importantly, the final G’ values observed in BG1 (~250 ± 1.26 Pa) and BG2 (~200 ± 0.64 Pa) fall within the range of storage moduli reported for native brain tissue in the literature [58]. The gelation profile of the organogel shows a rapid increase within the first 10 min, suggesting a highly efficient self-assembly process that is due to hydrophobic interactions and van der Waals forces. Beyond this point, the curve plateaus with the maximum G’ ~160 ± 0.98 Pa, indicating that the gelation process has reached completion, and substantial network formation.

Rheological properties and mechanical suitability of bigels for TBI applications.

**Figure 9:**
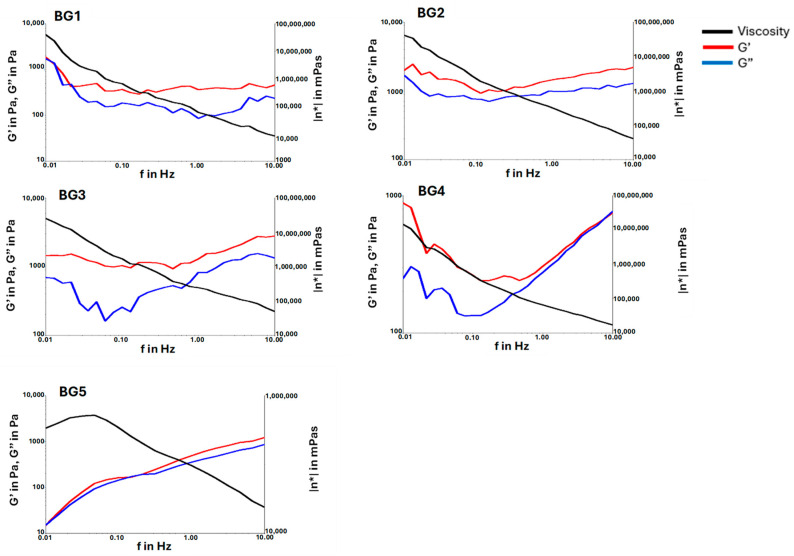
Storage and loss modulus, and viscosity of the bigels over a range of frequencies. * Represents the complex viscosity measured during the oscillatory frequency sweep test.

The rheological properties of the bigels (BG1 to BG5) were evaluated to assess their suitability for traumatic brain injury applications. This evaluation focused on measuring the storage modulus (G’, representing the elastic or solid-like behaviour) and loss modulus (G”, representing the viscous or liquid-like behavior) across a range of frequencies at 37.5 °C. Figure 9 illustrates the relationship between these moduli, providing insights into the mechanical stability and flow characteristics of the bigels under conditions relevant to biomedical applications.

In formulations BG1 to BG4, G’ consistently remains higher (dominates) than G” throughout the frequency spectrum. This indicates that these bigels exhibit solid-like, elastic behaviour, making them mechanically stable and resistant to deformation, especially at lower frequencies. This behaviour ensures that the bigel maintains its structure when applied to the injury site, indicating maintenance of predominantly elastic behaviour over the frequency range investigated.

Among the formulations, BG1 shows the highest G’ values, indicating it forms the most robust gel network. The high viscosity (as represented by the black curve) further implies a thick, gel-like consistency. This consistency may contribute to formulation retention following administration; however, retention was not evaluated in the present study. This characteristic may contribute to the release behaviour observed during in vitro testing.

As the organogel content increases from BG2 to BG5, the gap between G’ and G” narrows, particularly at higher frequencies. This indicates a decrease in elasticity, as the organogel phase becomes more prominent, resulting in a softer and less rigid gel network. However, at lower frequencies, the bigels still maintain mechanical stability, with high G’ values indicating strong structural integrity at these conditions. These properties indicate differences in mechanical response associated with changes in organogel content.

Finally, BG5, which has the highest organogel content, shows a crossover of G’ and G” at lower frequencies. This crossover suggests that BG5 behaves more like a viscous liquid than a solid, as G” dominates over G’. This transition suggests that BG5 is less mechanically stable than the other bigels, making it more fluid-like and offering higher flowability.

Overall, the combination of high storage modulus (~250 Pa), Young’s modulus (0.39 kPa), minimal oil leakage, porous microstructure, and predominantly elastic rheological behaviour distinguishes BG1 from the other formulations investigated. These properties indicate a mechanically stable hydrogel-dominant bigel with physicochemical characteristics that warrant further investigation as a localized biphasic drug-delivery platform. However, tissue retention, biological performance, and therapeutic efficacy remain to be established in future studies.

Conversely, BG5 exhibited lower mechanical stability and greater flowability owing to its higher organogel content. These characteristics may be advantageous in applications where enhanced deformability is desired, although injectability and in situ performance were not evaluated in the present study.

### 2.8. In Vitro Drug Release Profiles and Controlled Drug Delivery Potential of Bigels for TBI Therapy

Sustained drug release is considered advantageous in neurotherapeutic delivery strategies because it may maintain drug availability during periods associated with secondary injury processes following TBI which exhibits time-stratified therapeutic windows (acute: minutes to hours; subacute: hours to days; chronic: days to weeks). Based on this framework, a 72 h release window was chosen to span the acute and early subacute phases when secondary injury cascades (oxidative stress, inflammation, metabolic dysfunction) are most active, providing a rationale for evaluating release behaviour over a 72 h period [59,60]. A combination of NAC, TPGS, and nicotinamide has been proposed in previous studies as a multimodal therapeutic strategy because these compounds possess distinct biological activities relevant to neurotrauma. [9,61]. However, synergistic effects were not evaluated in the present study.

The calibration curves of the drugs, NAC, TPGS, and nicotinamide, were prepared with concentrations ranging from 1 μg/mL to 2.6 μg/mL for NAC, 5 μg/mL to 25 μg/mL for TPGS, and 5 μg/mL to 35 μg/mL for nicotinamide. The linear regression coefficients (R^2^) for all drugs were 0.9991, 0.9993, and 0.9955, respectively, indicating high linearity and accurate quantification across the tested range.

**Figure 10:**
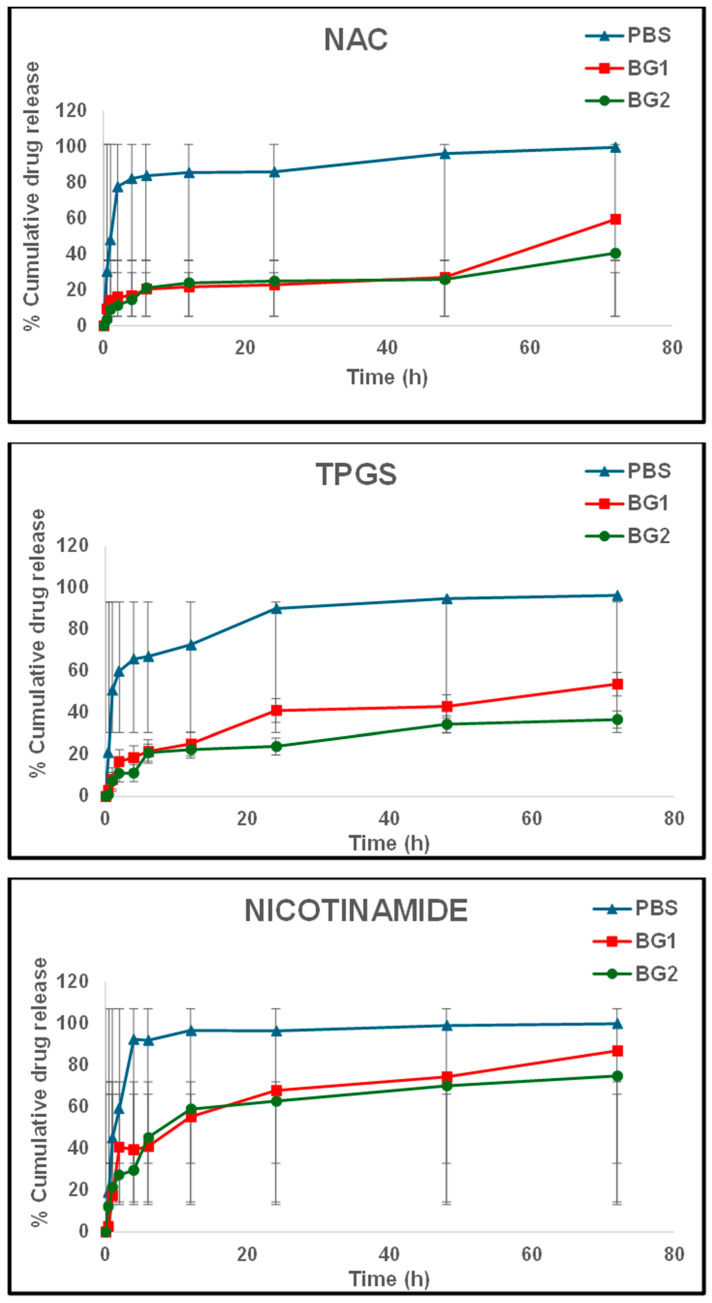
Graph depicting the release profiles of NAC, TPGS, and Nicotinamide from the bigels over time. Data are presented as mean ± standard deviation (SD), with error bars representing SD; *n* = 3.

As shown in Figure 10, drug release profiles were characterized by an initial rapid release phase (burst release) during the first 10 h, followed by a slower and sustained release phase. Nicotinamide shows the most rapid release among the drugs, reaching ~90% in PBS and ~60% in the bigel formulations, BG1 and BG2, during the 12 h phase (Figure 10). NAC demonstrated a ~30% release in BG1 during this period, slightly faster than in BG2, while TPGS showed an intermediate release profile (Figure 10).

The rapid release (burst phase) is commonly attributed to surface-adsorbed or loosely bound drug molecules in the matrix, while the subsequent slower release phase corresponds to drug diffusion through the hydrogel–organogel network. The initial release rates observed in bigels, compared to PBS, may be attributed to the presence of the organogel phase. For instance, the hydrophilic nature of nicotinamide may have facilitated its faster diffusion compared to NAC and TPGS, which interact differently with the methylcellulose/gelatin–organogel matrix due to their distinct physicochemical properties. Nicotinamide’s high aqueous solubility, combined with the continuous hydrogel network, may contribute to its more rapid release from the bigel matrix. In contrast, NAC and TPGS have different physicochemical properties. NAC (N-Acetylcysteine) is also hydrophilic but interacts differently due to its thiol and acetyl groups. These groups may form hydrogen bonds or interact with specific components in the methylcellulose/gelatin–organogel system, slightly slowing its release compared to nicotinamide. The TPGS (D-α-Tocopheryl Polyethylene Glycol 1000 Succinate) is amphiphilic, with hydrophilic and lipophilic characteristics. Its dual affinity likely causes partial partitioning into organogel domains, slowing diffusion relative to nicotinamide. Its polyethylene glycol (PEG) component makes it water-soluble, but its vitamin E-derived lipophilic region interacts with the hydrophobic organogel phase. This dual affinity can cause TPGS to be partially retained by the hydrophobic regions, leading to slower diffusion compared to nicotinamide.

The higher hydrogel content in BG1 promotes greater water absorption and swelling (63% swelling percentage), enhancing the diffusion of hydrophilic drugs such as NAC. In contrast, BG2, with a higher organogel content, shows slower drug release due to its reduced swelling (18% swelling percentage) and more hydrophobic matrix environment. These observations align with the leaching and swelling studies (Figure 6 and Figure S4) and support the proposed influence of hydrogel–organogel ratio on release behaviour.

The differing release profiles observed for nicotinamide, NAC, and TPGS demonstrate the capacity of the biphasic system to accommodate compounds with differing physicochemical properties. Nicotinamide exhibited more rapid release, whereas NAC and TPGS displayed comparatively sustained release behaviour. These differences are likely related to variations in aqueous solubility, molecular interactions with the matrix, and partitioning between hydrogel and organogel domains. The observed release behaviour reflects the functional performance of a dual-network polysaccharide–protein hydrogel system, where methylcellulose and gelatin act together to regulate diffusion pathways and modulate drug transport kinetics. This is consistent with the spirulina protein isolate nanogels (SG) incorporated into carboxymethyl chitosan based hydrogel system which resulted in slower release kinetics while single systems released drugs rapidly, and this trend can be seen in BG1 and BG2 with methylcellulose–gelatin hydrogel network [62]. This sustained release profile may be advantageous for future strategies intended to address prolonged oxidative stress and inflammatory processes following TBI. The formulation BG2 demonstrates a more controlled and sustained drug release profile for NAC and TPGS, providing a comparatively more sustained release profile for NAC and TPGS.

The drug release profiles highlight the potential of bigel systems to support differential release of compounds exhibiting distinct physicochemical properties; an initial burst release for immediate intervention, followed by sustained release to address prolonged oxidative stress and inflammation. These findings underline BG2 as the optimal formulation for controlled and sustained drug delivery, with BG1 exhibiting comparatively faster release, whereas BG2 demonstrated more sustained release behaviour. More importantly, compared with existing polysaccharide–protein hydrogel platforms, the present bigel system advances current designs by integrating a dual-network hydrogel phase with a biphasic organogel structure, enabling simultaneous modulation of diffusion, partitioning, and structural confinement, thereby providing a biphasic architecture that may offer greater flexibility in modulating diffusion and partitioning behaviour than conventional single-network hydrogel systems. Future studies should quantify drug loading and encapsulation efficiency to enable a more accurate assessment of drug retention and release kinetics from the bigel system.

The drug release mechanism of the two formulations was predicted by fitting the cumulative release data in three drug kinetic models: Kosmeyer-Peppas, Higuchi, and Weibull (Table 3). All formulations show the best fit for the Weibull model. The Higuchi model can be mathematically represented by Equation (2):

(2) $$f\left(x\right)=k\;\times \;t^{1/2}$$

where *f* is the fraction of cumulative drug release in time *t* and K is the Higuchian dissolution constant. K can also be described as a drug release rate based on diffusion: it indicates how quickly the drug diffuses out of the system matrix into the release medium, with higher K values representing faster rates [63]. BG1 exhibited higher K values than BG2, meaning that the drug release is faster in BG1 than in BG2. This could be attributed to the more porous structure of BG1 than BG2 (as evident in SEM studies), thus facilitating higher diffusion rates. It can also be noted again that through this model, nicotinamide is being released faster (burst phase as seen above) than NAC and TPGS because of its higher K values, and this may be due to the stronger interactions of NAC and TPGS with the matrix than nicotinamide. This observation means that the bigels can be categorized as matrix-type delivery systems [64].

The drug release mechanism was also predicted by fitting the cumulative release data into a Weibull model, which can be mathematically represented by Equation (3):

(3) $$m=1-\frac{{-(t-T_{0})}^{\beta }}{a}$$

where *m* is the fraction of the drug accumulated in solution at time *t*, a is the time scale parameter of the drug release process, *T* is the location parameter representing the lag time before the release can start, and *β* is the parameter characterizing the drug release graph (determines the release mechanism) [63]. The least squares regression method was used to fit this model using the regression add-in on Microsoft Excel 365 2017. The obtained *β* values of the drugs from each system were all <1, and this indicates a parabolic shape of the drug release curve, suggesting the diffusion-controlled release mechanism [65]. This suggests that drug transport is predominantly influenced by concentration-driven diffusion through the hydrated matrix through the hydrated hydrogel network and interconnected aqueous channels within the bigel matrix. Swelling of the methylcellulose–gelatin hydrogel phase likely plays a key role by increasing water uptake over time, thereby expanding diffusion pathways and facilitating drug mobility. In parallel, matrix relaxation and structural rearrangement of the polymer network may contribute to the sustained release profile, particularly as the system re-equilibrates following hydration.

The a values were higher in BG1 than in BG2, indicating a faster release of the drugs from BG1, as shown by the Higuchi model as well. It can also be noted that nicotinamide still shows faster release from the systems than the other two drugs, and this is a trend that has been observed throughout the drug release analysis. Generally, looking at the a and *β* values, it can be concluded that the higher content of organogel resulted in a slow and more controlled release.

The drug release mechanism was finally investigated by fitting the data into the Kosmeyer-Peppas (KP) model (Table 3). For both formulations, the release exponent, n (slope) of NAC and TPGS are <0.45, indicating that the release is primarily diffusion through the gel matrix (Fickian). In contrast, nicotinamide has n values of 0.45 and 0.46 in BG1 and BG2, respectively, indicating non-Fickian (anomalous) transport, which shows a combination of diffusion and slight polymer swelling effects. It can be concluded that diffusion is the dominant release mechanism, with nicotinamide showing a slightly faster initial release.

To sum up, given the bigel composition (hydrogel–organogel), an initial rapid release of nicotinamide through the hydrophilic hydrogel phase was supported by the Higuchi and KP model analyses. In contrast, the comparatively sustained release behaviour of NAC and TPGS may be associated with diffusion through and partitioning within the organogel-containing domains, as suggested by the Weibull and KP model analyses.

### 2.9. Evaluation of Cell Cytotoxicity of Bigels on PC12 via MTT Assay

**Figure 11:**
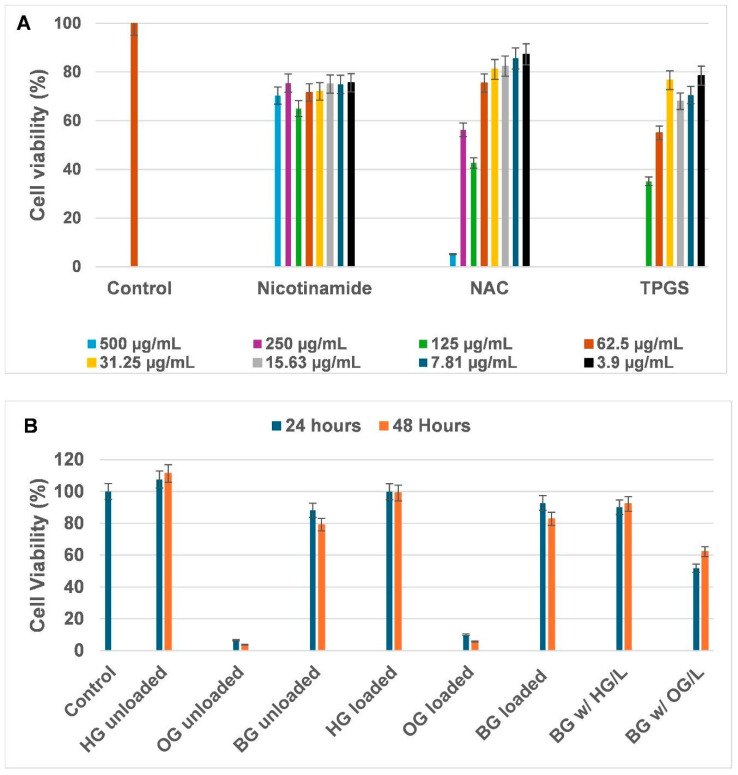
Cell viability assessment of PC12 cells treated with (**A**) varying concentrations (3.9–500 µg/mL) of nicotinamide, N-acetylcysteine (NAC), and D-α-Tocopherol polyethylene glycol 1000 succinate (TPGS) for 48 h. (**B**) with hydrogel (HG), organogel (OG), and bigel (BG) formulations, both unloaded and loaded with bioactives for 24 and 48 h. Data are presented as mean ± standard deviation (SD), and error bars represent SD.

An MTT assay was used to assess the cytocompatibility of both unloaded and loaded bigels (BG), hydrogel (HG), and organogel (OG) formulations on PC12 cells at 24 and 48 h. Only BG1 was used for this study. The concentrations of nicotinamide, N-acetyl-L-cysteine (NAC), and D-α-Tocopherol polyethylene glycol 1000 succinate (TPGS) used in the formulations were chosen based on preliminary MTT screening (Figure 11A), where the highest cell viability was observed at 500 µg/mL for nicotinamide, 62.50 µg/mL for NAC, and 31.25 µg/mL for TPGS. These concentrations were considered optimal for further incorporation into the respective formulations and cytocompatibility testing (Figure 11B) to ensure cell safety while maintaining concentrations suitable for subsequent formulation studies. The concentrations reflect ranges shown in previously investigated neurotrauma-related studies; however, the biological mechanisms associated with these compounds were not evaluated in the present work.

At both 24 and 48 h, loaded and unloaded HG formulations maintained cell viability above 90%, with no significant difference from the control group (*p* = 0.911 for loaded, *p* = 0.195 for unloaded HG, *n* = 3). This biocompatibility may be associated with the hydrated and polymer-rich nature of the hydrogel formulations as shown in Figure 8, the absence of a hydrophobic barrier, and the lack of an oxidative lipid phase. This high viability is consistent with the short-term stability of the hydrogel matrix, particularly at 24 h, where the material remained structurally intact with minimal swelling or degradation, as shown in Figure 6, which may have reduced exposure of cells to formulation-derived components during the early stages of incubation. The high cell viability can also be attributed to the biocompatible polymers used in the formulation, gelatin and methylcellulose. Gelatin is a denatured collagen derivative that offers multiple cell-recognition sites that aid cellular attachment and growth, while methylcellulose adds hydrophilicity and viscosity, thus promoting a favourable aqueous environment for nutrient and gaseous exchange [66,67]. In contrast, OG formulations (loaded and unloaded) showed low cell viability (<10%), with both formulations significantly different from the control group (*p* < 0.001). Several chemical and physical factors may have caused this low viability. Firstly, soybean oil is prone to lipid peroxidation, which has been reported to produce reactive oxygen species (ROS) and lipid peroxides that can induce oxidative stress and cell death [68]. Additionally, the dense and hydrophobic nature of the organogel may restrict nutrient and oxygen diffusion, leading to localized hypoxia and nutrient deficiency [69]. Furthermore, the lack of hydrophilic interfaces in this oil-rich matrix limits cell adhesion and signalling, preventing the cells from maintaining metabolic activity [70]. Collectively, these factors may contribute to a microenvironment that is less favourable for cell survival.

When the two systems were combined to create a bigel, a significant improvement in cell viability was observed compared to OG (Figure 11B). The unloaded bigel formulation showed cell viability with average values of ~88% and ~79% at 24 h and 48 h, respectively. Although some variability was observed among replicates, the overall trend consistently indicated enhanced cell metabolic activity compared to single-phase OG, suggesting that the bigel matrix supported acceptable PC12 cell viability under the conditions investigated. This observation may be associated with dilution of the organogel phase within the hydrogel matrix and reduced direct exposure of cells to lipid-rich domains. The bigel formulations maintained phase stability over the 48 h incubation period. The gel showed gradual degradation over the first 24 h, with minor fragmentation observable along the edges; however, no oil droplets or any sign of delayed demixing were noted at this stage. By 48 h, the gel had completely dissolved, yet the dissolution process did not result in visible oil migration or phase separation. This apparent dissolution was observed under cell culture conditions using DMEM, a nutrient-rich medium that can accelerate hydrogel swelling and erosion through polymer-solute interactions. In contrast, drug release studies were conducted in PBS, a simple ionic buffer that minimally interacts with the polymer network, thereby allowing sustained drug release to be maintained up to 72 h through diffusion from residual, microscopically dispersed organogel domains. Future work could measure the quantitative phase separation index to track microstructural stability and detect any subtle phase migration during degradation more precisely. No obvious evidence was observed to suggest that formulation breakdown adversely affected the viability measurements obtained during the study period. A significant difference from the control group was observed (*p* = 0.033). The loaded bigels exhibited slightly higher cell viability, with average values around 93% at 24 h and 83% at 48 h, and no significant difference from the control (*p* = 0.101), confirming that the incorporation of bioactives did not cause cytotoxicity. This trend may indicate that incorporation of the bioactives did not negatively affect cell viability; however, the mechanisms underlying this observation were not investigated. NAC, TPGS, and nicotinamide have previously been reported to possess antioxidant or cytoprotective properties in other experimental systems [71,72]. However, these effects were not evaluated in the present study. TPGS may also improve the solubility and cellular uptake of the bioactives within the bigel. These findings indicate that the loaded bigel maintained acceptable cytocompatibility under the conditions investigated. The study did not evaluate neuroprotective activity or therapeutic efficacy in a traumatic brain injury model. Therefore, further studies are required to determine whether the sustained delivery of NAC, nicotinamide, and TPGS translates into meaningful therapeutic effects. Mechanistic assays, including ROS inhibition and NGF-stimulated neurite outgrowth, as well as a TBI model, could be used in future studies to investigate their neuroprotective effects. PC12 cells provide a preliminary assessment of cytocompatibility but do not capture the complexity of the neural microenvironment, including neuron-glia interactions, inflammatory responses, or tissue-specific cellular behaviour. Thus, the current biological findings should be interpreted as preliminary cytocompatibility data rather than evidence of broader neurobiological compatibility. Future studies should therefore include more physiologically relevant cell types such as primary neurons, astrocytes, and microglia to better assess neurobiological responses.

**Figure 12:**
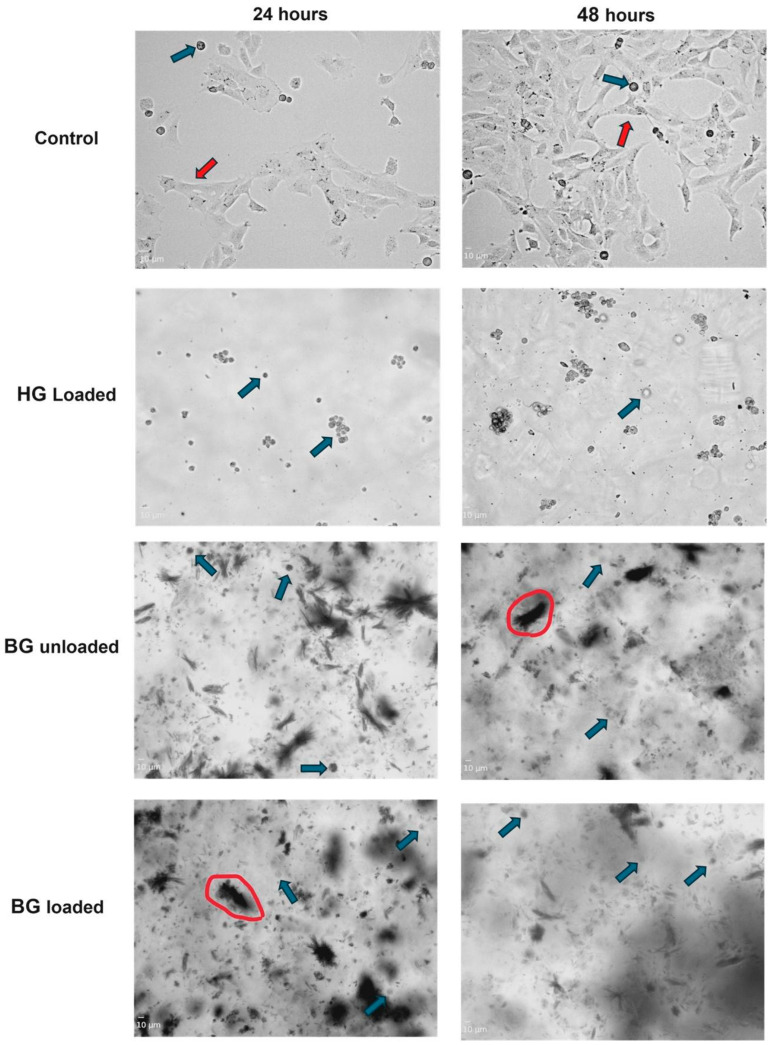
Microscopic images of PC12 cells seeded on bioactive-loaded bigel (BG) and hydrogel (HG), and unloaded bigel formulations at 24 and 48 h. Images were captured using CELENA^®^ S Digital Imaging System; three wells were analyzed per treatment (*n* = 3). Scale bar = 10 µm. Blue arrows indicate rounded, undifferentiated cells, while red arrows mark flattened cells exhibiting neurite-like morphological extensions. The red circular border annotates the debris likely associated with stearic acid from the organogel. Cell diameter and neurite length were measured from the same wells.

To evaluate morphology and cell attachment, PC12 cells were seeded onto bioactive-loaded hydrogel, bigel, and unloaded bigel formulations for 24 and 48 h (Figure 12). In control wells (containing only PC12 cells), a mixture of rounded and flattened cells with short, neurite-like extensions (length = 19.87 ± 2.34 µm) was observed at 24 h, indicating initial attachment. By 48 h, higher cell density and early neurite elongation (56.01 ± 18.02 µm) were apparent. These morphological features are typical of undifferentiated PC12 cells, which commonly show both rounded and flattened shapes in the absence of nerve growth factor (NGF) stimulation [73]. The presence of short neurite-like protrusions was observed morphologically [74]; however, differentiation was not assessed in this study. The average cell diameter was 10.05 ± 7.98 µm at 24 h and 14.13 ± 2.79 µm at 48 h, consistent with the viability measurements obtained in the control group. PC12 cells cultured on the loaded hydrogel maintained a predominantly rounded shape and remained viable, over 90%, as shown in Figure 11B, at both 24 and 48 h, with increased cell clustering over time. Quantitative analysis revealed cell diameters ranging from 8.67 to 12.48 µm (average 10.27 ± 1.32 µm) at 24 h and from 8.75 to 17.28 µm (average 12.06 ± 3.33 µm) at 48 h, similar to control cells. The lack of neurite extension indicates that while the hydrogel supports cell viability and the maintenance of cellular morphology under the conditions investigated, it does not strongly promote early differentiation under the tested conditions. The observed clustering and retained rounded morphology likely reflect the natural adhesive behaviour of PC12 cells combined with the hydrogel’s moderate stiffness and hydrophilic properties. As previously noted, gelatin provides cell-binding motifs that facilitate adhesion [75], thus supporting cell spreading and supporting its cytocompatibility within the scope of the present study.

The bigel formulations showed opacity toward the centre of the well, limiting visualization to the translucent periphery. Consequently, cell morphology and distribution within the interior regions of the constructs could not be verified, and the observed cellular responses may not fully represent the entire bigel, consistent with reported limitations in conventional viability-based assessments of opaque three-dimensional biomaterial systems [76]. Cells on the edges of both unloaded and loaded bigels remained rounded and sparsely distributed, with no visible neurite outgrowth. Cell diameters ranged from 7.90 to 14.93 µm (unloaded bigel) and 9.14 to 12.50 µm (loaded bigel) at 24 h, and from 11.12 to 13.08 µm (unloaded bigel) at 48 h (Figure 12). These measurements remained within ranges observed for viable PC12 cells under the experimental conditions used. Although peripheral fragmentation of the formulation components was observed, the results still indicated cell viability exceeding 70% relative to the hydrogel (Figure 11B). This observation may be associated with the thermal and enzymatic sensitivity of gelatin-based networks. and the low crosslink density at 0.1% genipin, combined with the small organogel content (5%) [77,78,79]. Mass loss studies also supported gradual matrix breakdown over 5 h (Table S2, BG1). The inclusion of methylcellulose may have contributed to maintaining overall structural integrity, while Tween 80, though improving interfacial stability, may have also plasticized the protein network and influenced phase redistribution over time. Neurite extensions were observed in control cells but not in hydrogel or bigel-treated groups. This absence of neuritogenesis is attributed to the lack of nerve growth factor (NGF) supplementation, as PC12 differentiation was not induced in this study. This may also reflect either the relative inertness of the matrices or suppression of differentiation due to matrix stiffness. The limited imaging depth represents a study limitation. Future work should therefore employ confocal z-stack viability staining to confirm cell survival throughout the full thickness of the bigel, rather than only at the translucent periphery. This approach, potentially combined with cryosectioning and cytoskeletal markers such as actin or β-III tubulin, would enable a comprehensive assessment of cell morphology and differentiation within the 3D matrix.

## 3. Conclusions

This study establishes the formulation feasibility of a stearate organogel–methylcellulose/gelatin bigel as a biphasic platform for localized neurotherapeutic delivery. The system was fabricated by dispersing a stearate-structured soybean oil organogel phase within a genipin-crosslinked methylcellulose/gelatin hydrogel network using hot emulsification, generating a hydrogel-dominant bigels with tunable phase architecture, viscoelastic behaviour, oil retention characteristics, and release kinetics.

Mechanistically, the methylcellulose/gelatin provided a hydrated continuous network, while genipin crosslinking contributed to network stabilization. The stearate organogel phase introduced lipid-rich domains capable of accommodating lipophilic compounds, whereas the aqueous hydrogel phase enabled incorporation of hydrophilic therapeutics. FTIR confirmed retention of characteristic functional groups from both phases, while XRD demonstrated reduced crystallinity and partial amorphization within the composite matrix, supporting successful formation of the biphasic system.

The hydrogel-dominant formulations exhibited compressive Young’s modulus values of 0.39–0.70 kPa and storage modulus values of approximately 200–250 Pa, which fall within reported ranges for soft brain tissue. These findings suggest mechanical relevance for future investigation in neurotherapeutic delivery applications, although direct brain-tissue compatibility remains to be established. Thermal transitions between 34.36 and 49.90 °C indicate matrix softening within a physiologically relevant temperature range, which may contribute to the observed release behaviour.

Variation in the hydrogel ratio significantly influenced microstructure, porosity, oil leakage, swelling behaviour, viscoelastic relaxation, and release kinetics. BG1 (95:5 hydrogel:organogel ratio), exhibited higher porosity and swelling, whereas BG2, with an 85:15 ratio, demonstrated lower porosity, greater structural density, minimal oil leakage, and more sustained release of NAC and TPGS. The release profiles were consistent with composition-dependent transport through hydrated and lipid-associated domains.

Taken together, the present bigel extends beyond existing polysaccharide–protein hydrogel platforms by combining a mechanically tunable and phase-stable methylcellulose–gelatin dual network with a structured organogel phase, thereby providing integrated control over network architecture, stability, viscoelastic behaviour, and release kinetics for therapeutics with differing physicochemical properties.

Both unloaded and drug-loaded bigels maintained >70% PC12 cell viability, supporting preliminary in vitro cytocompatibility. The improved viability of the composite bigels relative to organogel-only systems suggests that hydrogel-mediated phase confinement reduced direct lipid-domain exposure to cells while preserving lipophilic drug-loading capacity. However, these findings do not establish neuroprotective activity, therapeutic efficacy, or direct brain-tissue compatibility. Additional studies are required to evaluate. degradation behaviour, stability under physiologically relevant cerebrospinal fluid conditions, injectability, tissue retention, inflammatory responses, neuronal differentiation, and therapeutic performance in disease-relevant in vitro and in vivo models.

In conclusion, the stearate organogel–methylcellulose/gelatin bigel demonstrated phase stability, mechanical compliance, preliminary cytocompatibility, and multiphasic release of hydrophilic and lipophilic model therapeutics. These findings support further investigation of the platform as a candidate localized neurotherapeutic delivery system, while recognizing degradation, tissue interaction, retention, and therapeutic efficacy remain to be established.

## 4. Materials and Methods

### 4.1. Materials

Gelatin (Bloom 160, Type B, derived from bovine skin, pharmaceutical grade), methylcellulose (MC; Methocel^®^ MC; 27.5–32% as methoxyl content, viscosity range of 15–25 cP), Tween^®^ 80 (polysorbate 80, emulsifier, pharmaceutical grade), Genipin (natural crosslinking agent, ≥98% purity), Soybean oil (Soya oil from glycine max, food-grade), and Stearic acid (SA; 95%, pharmaceutical grade) were all purchased from Merck Chemicals Pty Ltd. (Germiston, South Africa). *N*-Acetyl-L-cysteine (NAC) (≥99% TLC, stored at +2 °C to +8 °C), Nicotinamide (≥98%, vitamin B3, store at room temperature), and D-α-Tocopherol polyethylene glycol 1000 succinate (TPGS) (BioXtra, water-soluble vitamin E conjugate, ≥98% purity, stored at +2 °C to +8 °C) were obtained from Merck Chemicals Pty Ltd. (Germiston, South Africa). Rat adrenal gland pheochromocytoma PC12 cells were purchased from Cellonex (Separations, Johannesburg, South Africa). Dulbecco’s Modified Eagles Medium-high glucose, trypsin-EDTA solution, Donor Equine Serum (DES), Gibco Fetal Bovine Serum, Trypan blue stain (0.4%), and Penicillin/Streptomycin/Amphotericin B (P/S/AB) solution were purchased from Gibco Invitrogen Corporation (Grand Island, NY, USA). Dimethyl sulfoxide (DMSO), hydrogen peroxide (H_2_O_2_), and Roche Cell Proliferation Kit I (MTT) were obtained from Sigma Aldrich (Steinheim, Germany). Double-deionized water was obtained from a Milli-Q system (Milli-Q, Millipore, Johannesburg, South Africa).

### 4.2. Methods

#### 4.2.1. Formulation

##### Preparation of the Organogel

Stearic acid (5% *w*/*v* relative to soybean oil) was used as the organogelator based on literature-reported critical gelation thresholds for stable self-assembled stearate networks [29]. Stearic acid was dissolved in soybean oil and heated to 70 °C under continuous magnetic stirring (15 min) until complete dissolution (Table 2). The molten mixture was then allowed to cool at room temperature (RT, 25 °C) without agitation to induce crystallization-driven network formation. Gelation occurred within approximately 20 min, after which the organogel was stabilized at 25 °C for an additional 60 min prior to further processing. Drug-loaded organogels were prepared in the same manner. For drug-loaded organogels, TPGS was incorporated into the molten organogel at 70 °C and stirred for a further 5 min to aid homogenous dispersion prior to cooling-induced gelation.

##### Preparation of Hydrogel

Gelatin (5% *w*/*v*) was dissolved in distilled water at 70 °C under constant stirring for 15 min. Methylcellulose was prepared using a temperature-induced dispersion method [80]. Briefly, half of the total solvent (distilled water) volume was heated up to 90 °C, and methylcellulose was gradually added under gentle agitation until fully wetted. The remaining cold water was added with continuous stirring to reduce the temperature to approximately 10 °C, facilitating complete polymer hydration and formation of a clear solution. Gelatin and MC solutions were combined to form a composite hydrogel system (Table 2). For drug-loaded hydrogels, nicotinamide and NAC were dissolved in the aqueous phase prior to polymer mixing to ensure molecular-level solubilization before network formation.

##### Preparation of the Bigels

Bigels were prepared via hot emulsification by combining the molten organogel phase with the MC-gelatin hydrogel phase at a controlled temperature. The organogel was liquefied at 70 °C and gradually introduced into the hydrogel phase containing Tween^®^ 80 (1% *v*/*v* relative to total formulation volume) as an emulsifier. This blend was homogenized using a rotor-stator homogenizer (Omni GLH850, Omni International Laboratory, Kennesaw, GA, USA) for 15 min at 5000 rpm until a homogeneous emulsion formed. Genipin (1% *v*/*v*, relative to hydrogel phase volume) was then added as a crosslinking agent and briefly homogenized for 10 s at 40 °C to initiate gelatin crosslinking while minimizing excessive shear-induced structural disruption. The resulting emulsified systems were carefully poured into 15 mL glass moulds and allowed to stabilize for 24 h at 25 °C to permit complete gelation and crosslink maturation prior to characterization. Scheme S1 illustrates the formulation workflow.

##### Freeze-Drying and Storage of Bigel Samples for Further Physicochemical and Physicomechanical Characterization

Freeze-drying was performed to preserve the internal microstructure for morphological and thermal analysis. Aliquots (1 mL) of freshly prepared bigels were transferred into a 12-well plate and pre-frozen at −80 °C in an ultra-low temperature freezer (Thermo Fisher Scientific, Waltham, MA, USA) for a minimum of 24 h to solidify the water content before freeze-drying. The frozen samples were transferred to a lyophilizer (Labconco FreeZone 2.5 L, Kansas City, MO, USA) and pre-cooled to −38 °C. The chamber pressure was reduced to 0.1 mbar, and the primary drying process was conducted at −38 °C for 24 h. This process removed free water while minimizing structural collapse due to capillary stress, ensuring that the samples remained dry and suitable for long-term storage and analysis.

##### Storage of Freeze-Dried Samples

Once the freeze-drying process was completed, the samples were removed from the lyophilizer and immediately transferred to airtight glass polytops, which were parafilm-sealed and stored with desiccant to avoid moisture reabsorption from the ambient environment. Samples were stored in an ultra-low temperature freezer at −78 °C to prevent residual moisture from causing degradation and ensure stability until further analysis. The freeze-dried samples were rehydrated with double-distilled water as needed for specific tests (such as biocompatibility) by carefully adding a pre-determined amount of water and allowing the bigels to equilibrate overnight at 4 °C.

#### 4.2.2. Microscopy

##### Microstructural Analysis via Bright-Field Microscopy

Brightfield microscopy was used to qualitatively assess macroscopic phase dispersion and homogeneity of hydrogel–organogel domains within hydrated formulations. Approximately 50–100 µL of each freshly prepared formulation was placed on a clean microscopic slide and allowed to set overnight under controlled conditions (refrigeration at 4 °C) to minimize evaporation and ensure consistent gelation conditions. Once the samples were set, a glass coverslip was carefully placed over the gels to avoid mechanical deformation and introducing air bubbles, which could interfere with imaging. The prepared slides were then visualized using a brightfield microscope (Olympus CX43 Optical CO. Ltd., Tokyo, Japan). The microstructure was analyzed at multiple magnifications (4×, 10×, 20×, and 40×). Images were captured under identical illumination settings for all formulations to enable comparative assessment. This technique was used solely for qualitative evaluation of phase continuity, dispersion uniformity, and presence of macroscopic heterogeneities.

##### Surface Morphology and Porosity Analysis via Scanning Electron Microscopy (SEM)

The surface morphology and porosity of the bigels were examined using a field emission scanning electron microscope (FEI Quanta 250 FEG, FEI Company, Hillsboro, OR, USA). To preserve the structural integrity of the bigels, the dry samples were mounted on aluminum stubs, using double-sided carbon tape to ensure proper adhesion. The freeze-dried samples were then sputter-coated twice with a 60/40 gold/palladium (AuPd) alloy to enhance conductivity and resolution, prevent charging, and facilitate imaging during electron microscopy. Samples were analyzed at an accelerating voltage of 10 kV under high vacuum conditions. The coating was applied using an EmiTech K550X Sputter coater (Quorum Technologies, Lewes, UK), yielding a 10 nm thick conductive layer. This thickness was selected to balance conductivity and surface resolution without excessively masking the samples’ morphological features. Micrographs were obtained at various magnifications (ranging from 500× to 5000×) to capture the pore structure, surface roughness, and overall morphology of the bigels. Image J software, version 1.54t (National Institutes of Health, Bethesda, MD, USA) was used to analyse the pore size and porosity of the resultant images. For each formulation, at least three independent images (*n* ≥ 3) were analyzed. Pore diameter was determined by measuring the longest internal dimension of individual pores, with a minimum of 50 pores quantified per formulation. Porosity (%) was determined by calculating the percentage of the image area occupied by pores based on threshold segmentation.

#### 4.2.3. Molecular Characterization Studies

##### Chemical Interaction Analysis via Fourier Transform Infrared (FTIR)

Fourier Transform Infrared Spectroscopy (FTIR) was conducted to identify molecular transitions and interactions of the lyophilized gel components (gelatin, methylcellulose, stearic acid, and incorporated drugs). Fourier transform infrared spectra were recorded using a PerkinElmer Spectrum Two FTIR spectrometer (PerkinElmer, MA, USA). The spectra studies were acquired over the wavenumber range of 4000–650 cm^−1^, with a resolution of 4 cm^−1^, an average of 20 scans/measurement under constant pressure of 120 psi, and a single-reflection diamond MIRTGS detector operating at a constant pressure of 120 psi to ensure consistent sample contact. Background spectra were recorded before sample measurements, and the data were processed using OriginPro software, version 8.0 to plot the FTIR spectra and analyze the characteristic peaks corresponding to specific functional groups, allowing for the identification of chemical interactions and potential shifts due to drug incorporation.

##### X-Ray Diffractometer (XRD)

To evaluate crystalline or amorphous states of incorporated drugs within the bigel matrix, a Rigaku Miniflex 60 X-ray diffractometer (XRD) (Rigaku Corporation, Tokyo, Japan) was used. Before analysis, the bigels were freeze-dried (as per Section Freeze-Drying and Storage of Bigel Samples for Further Physicochemical and Physicomechanical Characterization) and ground into a fine powder using a mortar and pestle to ensure homogeneity. The powdered samples were mounted onto the XRD sample holders and flattened out to prevent displacement during scanning. The analysis was performed at room temperature using a Cu-Kα radiation source (λ = 1.5406 Å), operating at 30 kV and 10 mA. The samples were scanned over a 2θ range of 5° to 90° at a scanning rate of 10° per minute. The diffraction patterns were analyzed to determine the crystalline components of the bigel, and any crystalline peaks were compared to standard reference materials to identify specific phases in the gel network. The crystallinity index was calculated using the following Equation (4). Peak integration was performed using OriginPro software following linear baseline correction. The amorphous halo was manually defined to distinguish crystalline peaks from the amorphous contribution prior to integration.

(4) $$Crystallinity\;Index\;\left(C_{i}\right)=\frac{Sum\;of\;areas\;under\;the\;crystalline\;peaks}{Total\;area\;under\;crystalline\;and\;amorphous\;peaks}$$

#### 4.2.4. Thermal Studies

The Mettler Toledo DSC1 STARe System (Schwerzenbach, Switzerland) was used to evaluate the thermal stability and transitions within the bigel formulations. The bigels were freeze-dried to obtain a powder form suitable for analysis. Pure aluminum pans were loaded with 5 to 8 mg of freeze-dried bigels and hermetically sealed with pierced aluminum lids to allow for pressure equalization during the analysis. An empty, sealed aluminium pan was used as a reference. The analysis was conducted over a temperature range of 20 °C to 150 °C with a heating rate of 2 °C/min under an inert nitrogen atmosphere. The nitrogen flow rate was maintained at 40 mL/min to ensure stable thermal conditions. Thermal transitions, such as melting points, glass transitions, and any exothermic or endothermic events, were recorded and analyzed to assess the thermal stability and potential phase transitions of the bigels.

#### 4.2.5. Stability Analysis via Leaching Studies

The stability of the bigels was evaluated by assessing the extent of oil leakage from the formulations in a humidity chamber at a controlled temperature of 37.5 °C, and 80% relative humidity as an accelerated stability condition for preliminary evaluation of formulation integrity and phase retention. The experiment was performed in triplicate (*n* = 3), and % leaching was reported as mean ± SD. A weighed sample of bigel (W_1_) was placed on a pre-weighed filter paper (Advantec^®^) (W_2_) and allowed to sit for 24 h at room temperature to facilitate any potential leaching of oil. After 24 h, the filter paper was re-weighed (W_3_), and the amount of leached oil was determined using Equation (5):

(5) $$Weight\;of\;leachate\;(W_{4})=W_{3}-W_{2}$$

The percentage of oil leaching was calculated using Equation (6):

(6) $$\%\;Leaching=\frac{W_{4}}{W_{1}}\times 100$$

This method provides insight into the oil retention capacity of the bigels, a key factor in their stability and integrity. Formulations with ≤10% leaching were considered stable based on published benchmarks [46].

#### 4.2.6. Swelling Behaviour Assessment via Equilibrium Weight Gain

The swelling behaviour of bigels was assessed using the equilibrium weight gain method [81]. The bigels were weighed (W_i_) and then immersed in 25 mL of PBS buffer solution (pH 7.4). Swelling was monitored with weight measurements taken at predetermined intervals (0 h, 30 min, 1 h, 2 h, 3 h, and 5 h) up to 5 h at 37 °C. At each interval, the samples were removed, blotted to remove excess buffer, and re-weighed (W_t_) to calculate the extent of swelling. The experiment was performed in triplicate (*n* = 3), and % swelling was reported as mean ± SD. The percentage swelling was determined using Equation (7):

(7) $$\%\;Swelling=\frac{{W_{t}-W}_{0}}{W_{0}}\times 100$$

where *W*_0_ is the first weight of the bigel, and *W_t_* is the weight at a specific time point. Swelling reflects the hydrophilic network’s capacity for fluid uptake, which influences mesh size, diffusion pathways, and mechanical softening under hydrated conditions. Excessive swelling may compromise mechanical stability, whereas controlled swelling contributes to conformability and drug diffusion regulation within soft neural environments.

#### 4.2.7. Mechanical Properties Analysis

The mechanical properties of the bigels were evaluated under compressive and oscillatory loading to quantify elastic modulus, viscoelastic relaxation, and gelation kinetics.

##### Unconfined Compression and Stress Relaxation Study

A uniaxial compression test was performed at 25 ± 2 °C using a static mechanical tester (TA.XTplus Texture Analyser Stable Microsystems, Godalming, UK). The experiment was done in triplicate (*n* = 3). Bigel formulations of uniform dimensions (height: 23.3 mm; diameter: 15.2 mm) were subjected to a stress relaxation test in their hydrated state using a flat-bottom probe (diameter: 12 mm (1/2”)). The probe applied a trigger force of 0.0294 N (equivalent to 3 g) to establish initial contact with the samples, after which it was advanced to a depth of 5 mm at a speed of 1 mm/s and held at constant strain at that position for 1 min to observe how the bigels relax over time, mimicking the mechanical behaviour of the brain under prolonged loading [29,45]. The relaxation behaviour provides information on the viscoelastic properties of the bigels, reflecting their mechanical stability. The stress–strain curve was derived from the stress relaxation data by plotting the initial stress response against the applied strain, assuming quasi-static conditions. Relaxation behaviour was evaluated by analyzing stress decay over time under constant strain. The slope of the initial linear region of this curve was used to estimate Young’s modulus of the bigel, representing its elastic stiffness (Figure S6). Reported values of the Young’a modulus of native brain tissue typically range from 0.1 to 1 kPa, depending on the brain region and testing method [52,53].

#### 4.2.8. Gelation Time Analysis

Dynamic non-destructive rheological analysis was performed using the ElastoSens^TM^ BIO2 (Rheolution Instruments, Montreal, QC, Canada) to assess the gelation time and storage modulus (G’) of the bigels. Notably, brain tissue typically exhibits storage modulus values ranging from 100 to 1000 Pa, depending on the region and measurement method, providing a physiological benchmark for comparison [52,58,82]. A sample of 2.5 mL of the formulation was added to the instrument’s elastic membrane sample holder 10 min after the addition of genipin. This waiting period allows the genipin crosslinking reaction to start uniformly throughout the sample, ensuring consistent distribution of the crosslinker. Additionally, this promotes homogeneity and reduces the likelihood of instrumental artefacts, thereby enhancing the accuracy and reproducibility of the gelation measurements. The measurement was conducted at 37 °C for 2 h (*n* = 3) to monitor the progression of gelation over time.

#### 4.2.9. Rheological Behaviour Assessment via Frequency Sweep Analysis

Rheological properties of the bigels and hydrogels were studied using a Haake MARS (Modular Advanced Rheometer System) (Thermo Electron Corporation, Karlsruhe, Germany) to assess their viscoelastic behaviour. A strain sweep test was first performed to determine the linear viscoelastic region (LVR), and subsequent frequency sweeps were conducted within this region (strain amplitude < 1%). A cone-plate sensor (sensor C35/1°, Ti), with a 35 mm diameter and a cone angle = 1°, was used with a 0.053 mm gap between the cone and plate. Oscillatory frequency sweep analysis was conducted over a frequency range of 0.01–10 Hz with constant strain, within a range of 20–50 °C to simulate hyperthermic stress. The test was done in triplicate (*n* = 3). The RheoWin PC Software v3 was used for data collection and analysis, providing insights into the frequency-dependent rheological properties of the bigels. The storage modulus (G’), loss modulus (G”), and the bigels’ crossover points were interpreted to evaluate the transition between elastic and viscous behaviour.

#### 4.2.10. In Vitro Drug Release Studies

The in vitro drug release profile of the bigels was evaluated in PBS (pH 7.4) at 37 °C. An accurately weighed drug-loaded bigel sample was placed inside a snake’s skin dialysis bag (molecular weight cutoff 3.5 kDa, pre-soaked in PBS). The dialysis bag was immersed in 50 mL of PBS buffer at 37 °C with gentle stirring (~50 rpm) to facilitate diffusion and maintain a constant temperature. At predetermined time intervals (0 h, 30 min, 1 h, 2 h, 4 h, 6 h, 12 h, 24 h, 48 h, and 72 h), 3 mL of the release medium was withdrawn and immediately replaced with an equal volume of fresh PBS to maintain sink conditions. Sink conditions were validated by ensuring that the drug concentration in the release medium did not exceed 10% of their solubility in PBS, particularly for hydrophobic drugs such as TPGS. This replacement ensured that a sufficient concentration gradient was maintained throughout the study. The concentration of released drugs (NAC, nicotinamide, and TPGS) in the withdrawn samples was quantified using a nanophotometer (Implen Nanophotometer^®^ NP80, Munich, Germany). Calibration curves for each drug (λ_max_ NAC = 202 nm, TPGS = 282 nm, and nicotinamide = 262 nm) were prepared in PBS over a range of concentrations to ensure accuracy, with regression coefficients (R^2^ > 0.995) confirming linearity (Figure S7). PBS was used as the blank for background subtraction, and all absorbance measurements were automatically corrected for pathlength by the instrument, with calibration curves validated to confirm the absence of spectral interference at the selected wavelengths. The cumulative percentage drug release was calculated as the cumulative amount of drug released at each sampling time relative to the theoretical amount of drug initially incorporated into the formulation. All release studies were performed in triplicate (*n* = 3) to ensure reproducibility, and results were expressed as the cumulative percentage of drug released over time.

#### 4.2.11. In Vitro Biocompatibility via Cytotoxicity Assays (MTT Assay)

The biocompatibility of the bigel formulations was evaluated through cytotoxicity assays conducted on the rat adrenal pheochromocytoma PC12 mixed adherent cell line from Cellonex (Separations, Johannesburg, South Africa), which is critical for modelling the cellular environment of the brain [83]. The cytotoxicity study aimed to determine the impact of bigels exposure on cell viability, providing insights into their suitability for traumatic brain injury.

The PC12 cells were cultured in tissue culture-treated (TPP) T-75 flasks using Dulbecco’s Modified Eagles Medium (DMEM) supplemented with 15% FBS, and 1% P/S solution in a humid 5% CO_2_ atmosphere at 37 °C to about 80% confluence. The growth medium was changed every 48 h after washing with PBS to remove dead cells. For seeding, PC12 cells were first detached from the T-75 flask using 2.5 mL of trypsin and then neutralized with an equal volume of media. The suspension was centrifuged for 5 min at 300× *g*, and the pellet was resuspended in 3 mL of fresh medium. 10 µL of trypan blue dye was added to 10 µL of the cell suspension and left for 5 min for a full reaction with the cells before counting. A 10 µL of the trypan blue-cell suspension mixture was loaded into each of the two chambers of a clean LUNA-II slide and counted using the LUNA-II automated cell counter (Logos Biosystems, Anyang, South Korea). PC12 cells were seeded at a density of 1 × 10^4^ cells/mL (100 µL) onto the treatment and incubated for 24 and 48 h at 37 °C with an atmosphere of 5% CO_2_. A seeding density of 1 × 10^4^ cells/mL was chosen based on optimization studies, ensuring confluency without overgrowth.

The treatment: 20 µL of bioactive-loaded hydrogels, organogels, bigels, and unloaded hydrogels, organogels, and bigels were added to each well of a 96-well plate and allowed to set for 15–20 min with the lid closed. Each well corresponds to ~0.321 cm^2^ surface area, yielding a gel surface area-to-cell ratio of 0.321 cm^2^ per 10^4^ cells. In the bioactive-loaded formulations, this translates to 10 µg nicotinamide, 1.25 µg N-acetylcysteine (NAC), and 0.625 µg TPGS per well. The plate was then sterilized under UV for 10 min before seeding. Pure bioactive treatments were made by dissolving the bioactive in DMEM to make different concentrations ranging from 3.16 to 500 µg/mL for individual drugs. This was done in triplicate.

Controls:

- Untreated cells were used as the positive control group (baseline for 100% viability).
- DMSO was added to the cells as the negative control. The final concentration of DMSO used was 10% *v*/*v*, which demonstrated its cytotoxic effects on PC12 cells, as there was reduced metabolic activity after the MTT assay.

Blanks (no cells):

- Media only (background absorbance of medium/ baseline subtraction),
- Media with hydrogels, organogels, and bigels (checks optical interference of matrix),
- Media with pure bioactives were used to assess baseline coloration and absorbance correction.

An MTT assay was employed to measure cell viability [83,84]. The assay is based on the reduction of MTT (a yellow tetrazolium salt) to formazan crystals by metabolically active cells, providing a quantitative measure of cellular metabolic activity. A 10 µL aliquot of a ready-to-use 0.5 mg/mL MTT solution (used as provided by the manufacturer, without further preparation) was added to each well, and the cells were further incubated for 4 h. To minimize light exposure, all procedures were performed under a laminar flow unit with reduced lighting, and the plate was covered with aluminum foil during incubation. The solubilizing agent (100 µL, DMSO), which dissolves formazan crystals, was then added, and the cells were incubated overnight. The supernatant in each well was transferred to a new 96-well plate to avoid any inaccuracy in absorbance readings from the debris of the bigel. The absorbance of dissolved formazan crystals in the 96-well plates was measured at 570 nm, with background subtraction at 690 nm, using a multi-plate reader (Biotek, Winooski, VT, USA). The background absorbance correction also included bigel/drug in media without cells to exclude interference. All MTT assays were performed in triplicate. The darker the solution, the greater the number of metabolically active viable cells. Higher absorbance values indicate greater cell viability. The % cell viability of treated PC12 was calculated using Equation (8):

(8) $$\%\;cell\;viability=\frac{Average\;absorbance\;of\;treated\;cells-blank}{Average\;absorbance\;of\;untreated\;cells-blank}\times 100$$

where

Untreated control = PC12 cells + media only.

Blank = media + samples or drugs without cells.

The results were statistically compared with untreated control cells to assess potential cytotoxic effects, and absorbance values greater than the control positive indicated cell viability and increased metabolic activity.

The baseline morphology and attachment of PC12 cells were assessed using the loaded and unloaded bigels and hydrogel, which were chosen based on their non-cytotoxicity observed in the preliminary MTT assay. Briefly, 1 mL of PC12 cells was seeded at a density of 5 × 10^4^ directly into 12-well plates onto 100 µL of sterile hydrogels, organogels, and bigels in culture medium and incubated for 24 and 48 h at 37 °C with an atmosphere of 5% CO_2_. Untreated cells were used as the positive control group (baseline for 100% viability), and DMSO was added to the cells as the negative control. The final concentration of DMSO used was 10% *v*/*v*. This was done in duplicates. Phase-contrast imaging was performed using the CELENA^®^ S Digital Imaging System (Logos Biosystems). For each well of the 12-well plate, images were captured at 10× and 20× objectives. The 10× objective provided full-well coverage to assess overall cell distribution, while the 20× objective enabled detailed evaluation of single-cell morphology, including cell shape, spreading, and confluence.

#### 4.2.12. Statistical Analysis

All results were expressed as mean ± standard deviation with biological replicates on *n* = 3 per group. Statistical significance was determined using a variety of tests, including a two-sample t-test for comparing treated and untreated cells, one-way ANOVA for comparing the responses of multiple treatment groups, and time-series analysis for studying the effects of treatment over time. A 95% confidence interval was used, and a *p*-value less than 0.05 was considered statistically significant. Statistical analysis was performed using SigmaPlot, version 15.0 (Systat Software Inc., San Jose, CA, USA), utilizing its built-in statistical functions.

## Figures and Tables

**Table 1:** Filter paper mass gain and percentage oil leaching of the formulations (*n* = 3).

Sample	Mass of Filter Paper (Without Bigel) (g) 0 h	Mass of Filter Paper (Without Bigel) (g) 24 h	Mass Gained (g) 24 h	% Leaching
BG 1	1.5345 ± 1.15 × 10^−4^	1.5363 ± 1.15 × 10^−4^	0.0018 ± 5.77 × 10^−5^	0.04 ± 1.42 × 10^−3^
BG 2	1.5236 ± 1.15 × 10^−4^	1.5301 ± 5.77 × 10^−5^	0.0065 ± 1.00 × 10^−4^	0.20 ± 2.47 × 10^−3^
** BG2A	1.5229 ± 5.57 × 10^−5^	1.5301 ± 5.77 × 10^−5^	0.0072 ± 0.00	0.18 ± 0.00
** BG2B	1.5363 ± 7.73 × 10^−3^	1.5401 ± 5.77 × 10^−3^	0.0038 ± 1.97 × 10^−3^	0.12 ± 4.91 × 10^−2^
** BG2C	1.5233 ± 1.52 × 10^−4^	1.5300 ± 5.83 × 10^−3^	0.0067 ± 5.97 × 10^−3^	0.25 ± 1.48 × 10^−1^
BG 3	1.5328 ± 5.77 × 10^−5^	1.5449 ± 2.08 × 10^−4^	0.0121 ± 2.31 × 10^−4^	0.29 ± 5.61 × 10^−3^
BG 4	1.5296 ± 2.89 × 10^−4^	1.5688 ± 1.53 × 10^−4^	0.0392 ± 2.00 × 10^−4^	0.96 ± 4.89 × 10^−3^
BG 5	1.5322 ± 1.53 × 10^−4^	2.9822 ± 1.15 × 10^−4^	1.4500 ± 1.00 × 10^−4^	35.8 ± 2.47 × 10^−3^

** Bigels containing Nicotinamide, NAC, and TPGS (2A: 10 mg, 2B: 20 mg, and 2C: 50 mg).

**Table 2:** Composition and coding of the different organogels, hydrogels, and bigels formulated with different ratios of organogel to hydrogel and different hydrogel structures.

Sample	Organogel (5% *v*/*v*)		Hydrogel (5% *v*/*v*)			Tween 80 (1% *v*/*v*) (mL)	Genipin 1% *v*/*v*) (mL)
	Soybean Oil (mL)	Stearic Acid (g)	Gelatin (g)	Methylcellulose (g)	Distilled Water (mL)		
OG	5.0	0.25	-	-	-	-	-
HG 1	-	-	0.5	-	10.0	-	-
HG 2	-	-	0.5	-	9.9	-	0.1
HG 3	-	-	-	0.5	10.0	-	-
HG 4	-	-	-	0.5	9.9	-	0.1
HG 5	-	-	0.5	0.5	10.0	-	-
HG 6	-	-	0.5	0.5	9.9	-	0.1
BG 1	1.0	1.0	1.0	1.0	18.6	0.2	0.2
BG 2	3.0	1.0	1.0	1.0	16.6	0.2	0.2
** BG2A	3.0	1.0	1.0	1.0	16.6	0.2	0.2
** BG 2B	3.0	1.0	1.0	1.0	16.6	0.2	0.2
** BG 2C	3.0	1.0	1.0	1.0	16.6	0.2	0.2
BG 3	5.0	1.0	1.0	1.0	14.6	0.2	0.2
BG 4	7.0	1.0	1.0	1.0	12.6	0.2	0.2
BG 5	10.0	1.0	1.0	1.0	9.6	0.2	0.2

** Bigels containing different amounts of Nicotinamide, NAC, and TPGS (BG2A: 10 mg of each, BG2B: 20 mg of each, and BG2C: 50 mg of each).

**Table 3:** Drug release mechanisms of BG1 and BG2 formulations.

Formulation	Drug	Weibull (R^2^)	Higuchi (R^2^)	Best Fit	KP Model	
					*n*	Type of Flow
BG1	Nicotinamide	0.993	0.858	Weibull	0.45	Non-Fickian
	TPGS	0.993	0.927	Weibull	0.40	Fickian
	NAC	0.994	0.806	Weibull	0.27	Fickian
BG2	Nicotinamide	0.993	0.868	Weibull	0.46	Non-Fickian
	TPGS	0.993	0.909	Weibull	0.37	Fickian
	NAC	0.999	0.878	Weibull	0.32	Fickian

## Data Availability

The data presented in this study are available upon request from the authors.

## Supplementary Materials

The following supporting information can be downloaded at: https://www.mdpi.com/article/10.3390/gels12070574/s1. Figure S1: Representative photographs of the organogel (OG) and hydrogel formulations (HG1, HG2, HG3, HG4, HG5, and HG6) following gelation; Figure S2: *p*-values of the leaching area of the bigels; Figure S3: Brightfield micrographs of organogel (OG), hydrogels (HG1, HG6), and BG1 to BG5 visualized at 10× magnification (100 µm), illustrating microstructural organization and phase distribution of the formulations; Figure S4: Bar graph showing the percentage of oil leaching from free bigels and drug-loaded bigels (BG2A: 10 mg, BG2B: 20 mg, BG2C: 50 mg) after 24 h 37 °C and 80% relative humidity as a preliminary formulation stability assessment. Bars are labelled with different lowercase letters (a–e) to indicate statistically significant differences between formulations. Bars with the same letter are not significantly different. Bars with different letters are significantly different (*p* < 0.001), and error bars represent mean ± SD; Figure S5: FTIR data of BG1–BG5 before (left) and after (right) swelling studies; Figure S6: Stress-Strain curves of BG1 and BG2; Figure S7: Calibration curves for NAC, nicotinamide, and TPGS were done in phosphate-buffered saline (PBS; pH 7.4) at 37 °C by measuring a series of known concentrations (NAC: 0–2.6 µg/mL, nicotinamide: 0–35 µg/mL, and TPGS: 0–35 µg/mL) using a nanophotometer (Implen Nanophotometer^®^ NP80, Munich, Germany) at a wavelength of 202, 262, 282 nm for NAC, nicotinamide, and TPGS, respectively. The resulting calibration curves were constructed by plotting absorbance values on the y-axis against corresponding concentrations (µg/mL) on the x-axis. A linear regression analysis, constrained to pass through the origin (intercept = 0), yielded a correlation coefficient (R^2^) of 0.9991, 0.9931, and 0.9993 for the drugs, respectively, indicating strong linearity; Scheme S1: Graphical representation of the formulation of hydrogels, organogels, and bigels; Table S1: XRD parameters of bigels with reference to stearic acid; Table S2: Weight loss measurements of bigels at 37 °C (*n* = 3).
