# Supplementary material for: Formulation Feasibility of a Mechanically Compliant Stearate Organogel–Methylcellulose/Gelatin Bigel for Localized Neurotherapeutic Delivery
Source: Gels. 2026 Jun 29;12(7):574. doi: 10.3390/gels12070574 (PMC13409561; doi:10.3390/gels12070574)
Supplement: Supplementary file 1 [file gels-12-00574-s001.zip › gels-4376852-supplementary.pdf]

Supplementary data

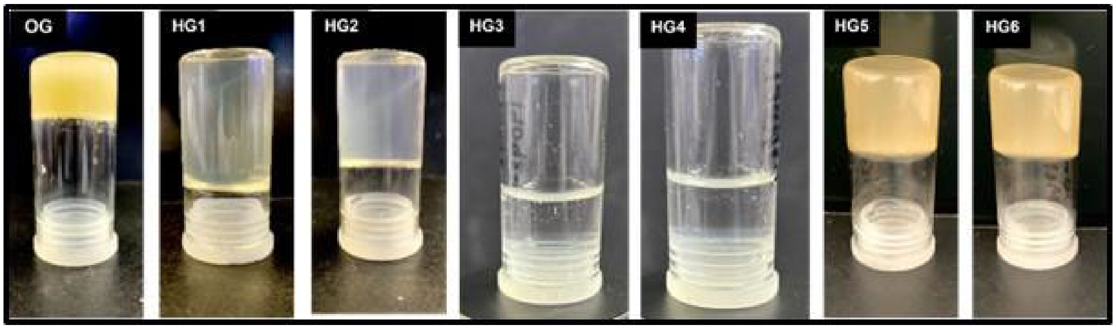

Figure S1: Representative photographs of the organogel (OG) and hydrogel formulations (HG1, HG2, HG3, HG4, HG5, and HG6) following gelation.

One Way Repeated Measures Analysis of Variance Tuesday, September 30, 2025, 11:46:49

Data source: Data 1 in Notebook2

Normality Test (Shapiro-Wilk) Passed (P = 0,948)

Equal Variance Test: Passed (P = 0,269)

Treatment Name	N	Missing	Mean	Std Dev	SEM
BG1	3	0	0,670	0,0608	0,0351
BG2	3	0	1,067	0,104	0,0601
BG3	3	0	2,200	0,200	0,115
BG4	3	0	3,103	0,105	0,0606
BG5	3	0	7,273	0,261	0,151

Source of Variation	DF	SS	MS	F	P
Between Subjects	2	0,0220	0,0110		
Between Treatments	4	83,953	20,988	684,253	<0,001
Residual	8	0,245	0,0307		
Total	14	84,221			

The differences in the mean values among the treatment groups are greater than would be expected by chance; there is a statistically significant difference (P = <0,001). To isolate the group or groups that differ from the others use a multiple comparison procedure.

Power of performed test with alpha = 0,050: 1,000

All Pairwise Multiple Comparison Procedures (Holm-Sidak method):
Overall significance level = 0,05

Comparisons for factor:

Comparison	Diff of Means	t	P	P<0,050
BG5 vs. BG1	6,603	46,177	<0,001	Yes
BG5 vs. BG2	6,207	43,403	<0,001	Yes
BG5 vs. BG3	5,073	35,478	<0,001	Yes
BG5 vs. BG4	4,170	29,161	<0,001	Yes
BG4 vs. BG1	2,433	17,016	<0,001	Yes
BG4 vs. BG2	2,037	14,242	<0,001	Yes
BG3 vs. BG1	1,530	10,699	<0,001	Yes
BG3 vs. BG2	1,133	7,925	<0,001	Yes
BG4 vs. BG3	0,903	6,317	<0,001	Yes
BG2 vs. BG1	0,397	2,774	0,024	Yes

Figure S2: P-values of the leaching area of the bigels.

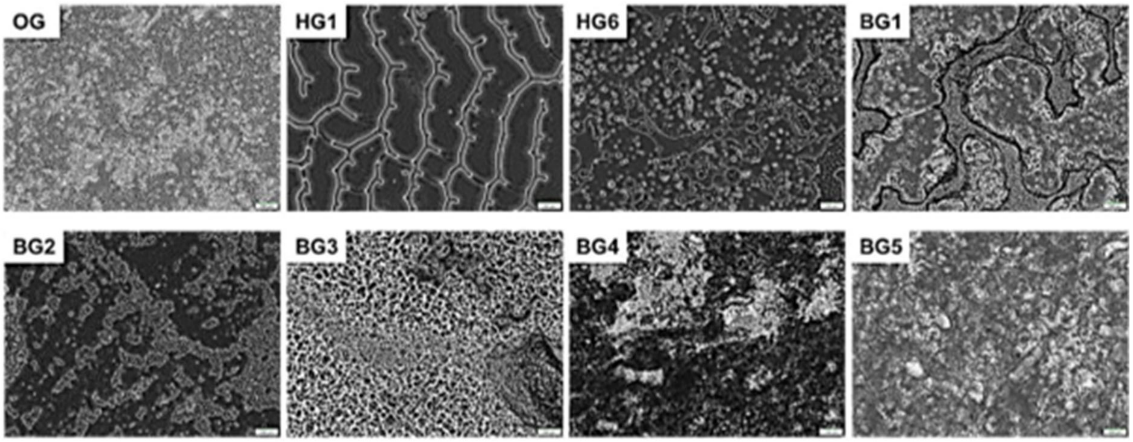

Figure S3: Brightfield micrographs of organogel (OG), hydrogels (HG1, HG6), and BG1 to BG5 visualized at 10x magnification (100 μm), illustrating microstructural organization and phase distribution of the formulations.

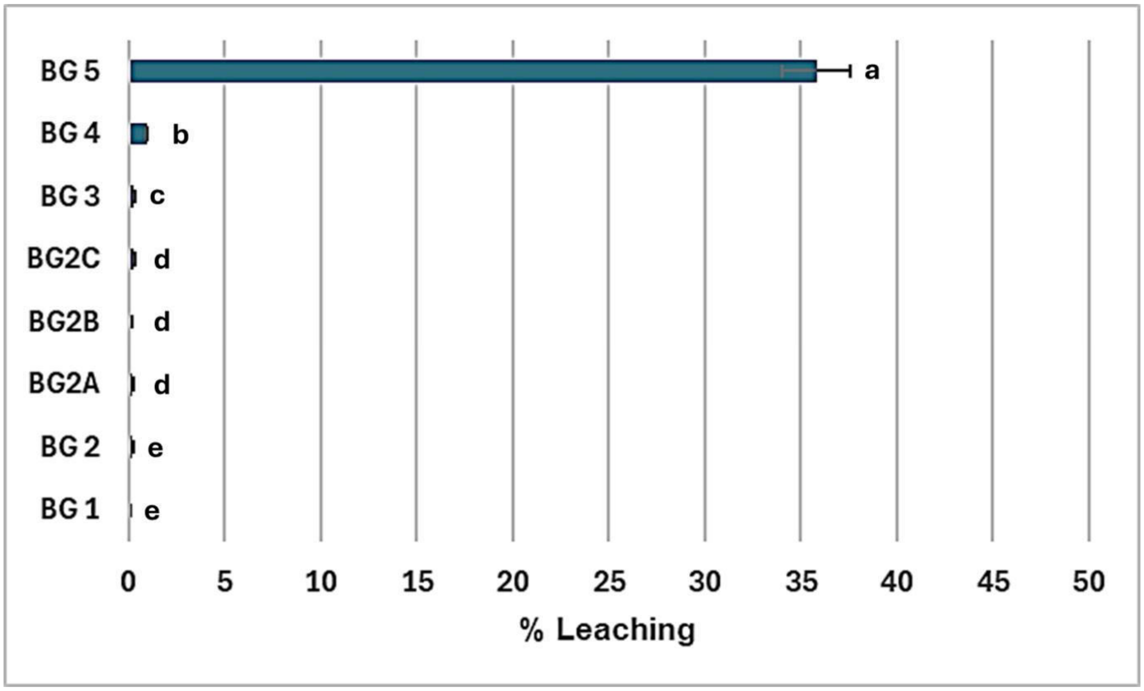

Figure S4: Bar graph showing the percentage of oil leaching from free bigels and drug-loaded bigels (BG2A: 10 mg, BG2B: 20 mg, BG2C: 50 mg) after 24 hours 37°C and 80% relative humidity as a preliminary formulation stability assessment. Bars are labelled with different lowercase letters (a-e) to indicate statistically significant differences between formulations. Bars with the same letter are not significantly different. Bars with different letters are significantly different ($p < 0.001$), and error bars represent mean ± SD.

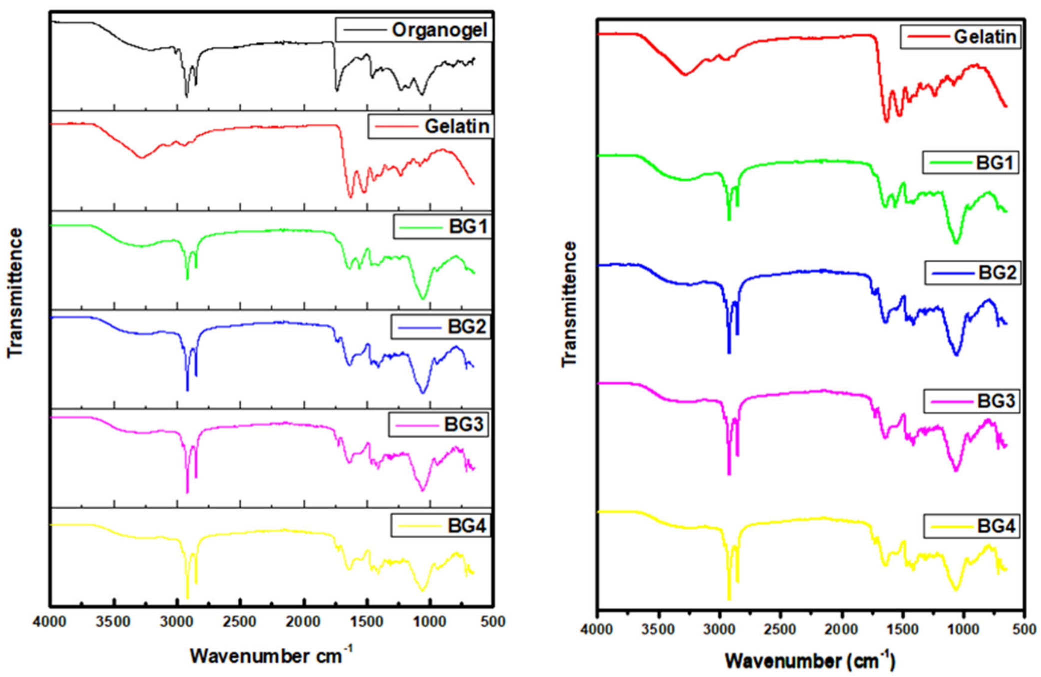

Figure S5: FTIR data of BG1 – BG5 before (left) and after (right) swelling studies.

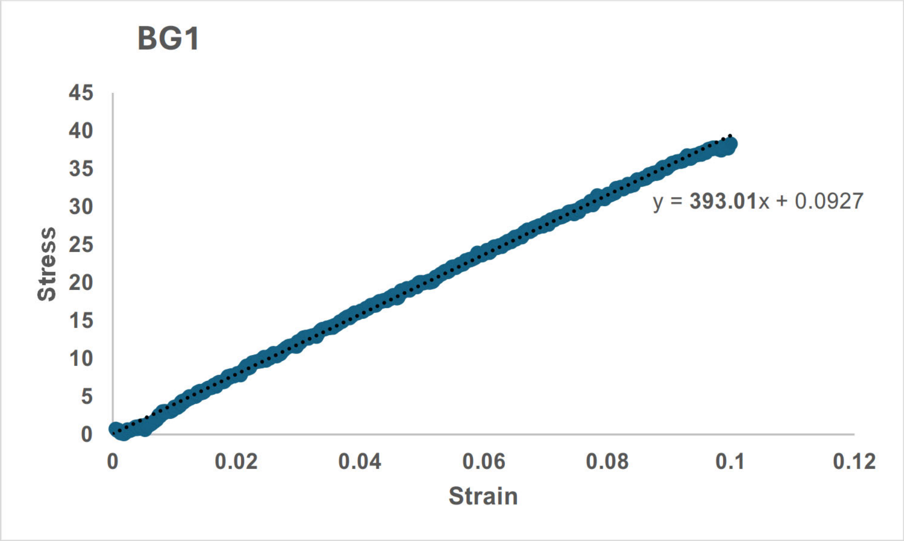

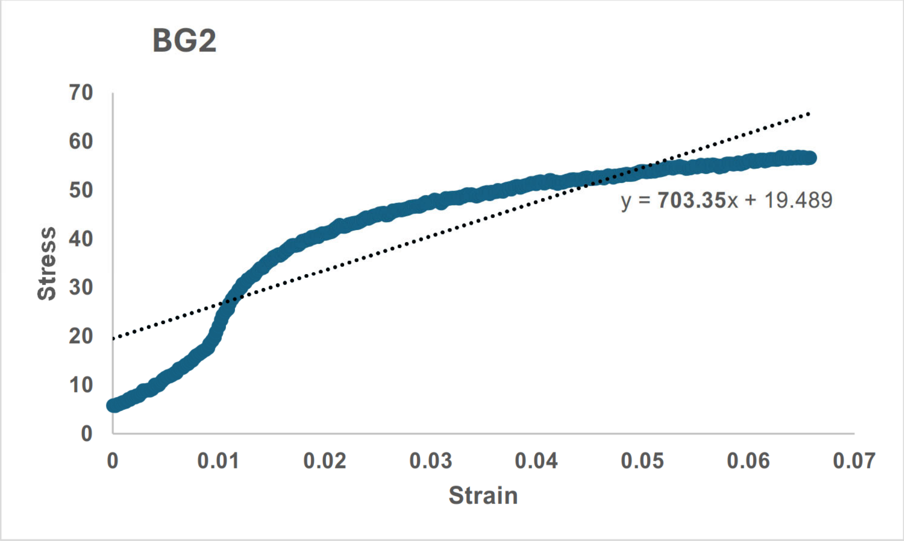

Figure S6: Stress-Strain curves of BG1 and BG2

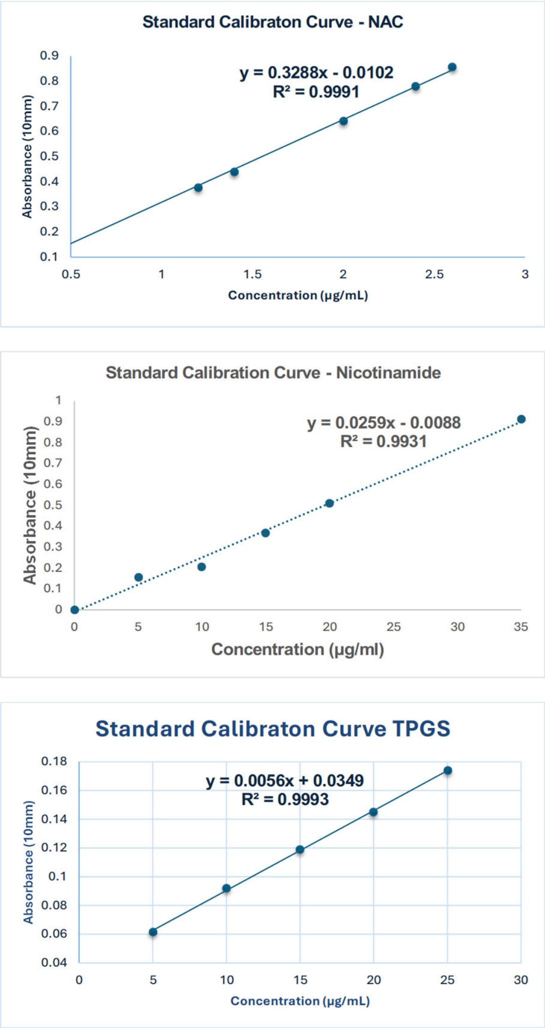

Figure S7: Calibration curves for NAC, nicotinamide, and TPGS were done in phosphate-buffered saline (PBS; pH 7.4) at 37 °C by measuring a series of known concentrations (NAC:0–2.6 µg/mL, nicotinamide:0-35 µg/mL, and TPGS:0-35 µg/mL) using a nanophotometer (Implen Nanophotometer ® NP80, Germany) at a wavelength of 202, 262, 282 nm for NAC, nicotinamide, and TPGS, respectively. The resulting calibration curves were constructed by plotting absorbance values on the y-axis against corresponding concentrations (µg/mL) on the x-axis. A linear regression analysis, constrained to pass through the origin (intercept = 0), yielded a correlation coefficient (R^2) of 0.9991, 0.9931, and 0.9993 for the drugs, respectively, indicating strong linearity.

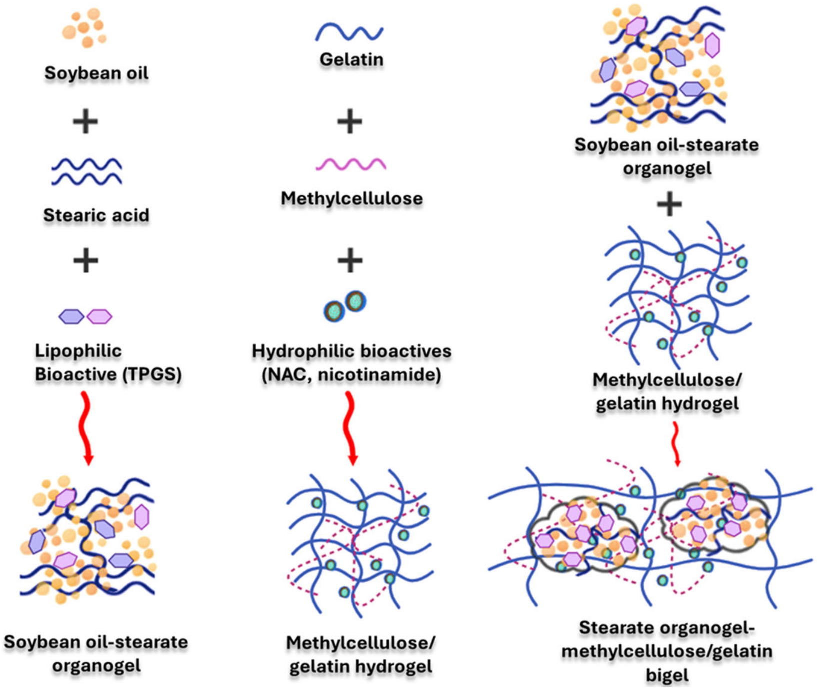

Scheme S1: Graphical representation of the formulation of hydrogels, organogels, and bigels.

**Table S1:** XRD parameters of bigels with reference to stearic acid					
**Sample**	**2θ**	**d-spacing (Å)**	**Area under the crystalline peaks**	**Total area under the crystalline and amorphous peaks**	**C_i**
**OG**	21.42	**4.14**	895.66	1226.18	**0.73**
**BG1**	20.11	**4.41**	186.11	506.66	**0.37**
**BG2**	19.94	**4.45**	215.12	739.12	**0.29**
**BG3**	19.50	**4.55**	187.53	764.61	**0.25**
**BG4**	19.59	**4.53**	161.18	661.29	**0.24**
**BG5**	19.54	**4.55**	159.07	804.42	**0.20**

**Table S2:** Weight loss measurements of bigels at 37°C (n = 3)						
Bigels	W_0	$W_{0.5hr}$	W_{1hr}	W_{2hr}	W_{3hr}	W_{5hr}
BG1	0.7691 ± 2.00×10^{-4}	1.2634 ± 5.77×10^{-5}	1.0912± 5.77×10^{-5}	1.1045 ± 5.77×10^{-5}	1.0004 ± 5.77×10^{-5}	0.8772 ± 5.77×10^{-5}
BG2	1.4147 ± 6.46×10^{-4}	1.6353 ± 3.82×10^{-2}	1.5935 ± 5.63×10^{-3}	1.5866 ± 5.72×10^{-3}	1.4524 ± 6.73×10^{-3}	1.2446 ± 5.00×10^{-3}
BG3	1.2559 ± 5.16×10^{-3}	1.5190 ± 8.39×10^{-3}	1.3994 ± 9.12×10^{-3}	1.3236 ± 2.90×10^{-3}	1.0525 ± 2.12×10^{-3}	0.8598 ± 7.01×10^{-3}

BG4	1.8357 ± $4.15x10^{-3}$	1.9159 ± $4.52x10^{-3}$	1.7836 ± $4.77x10^{-3}$	1.5328 ± $5.41x10^{-3}$	1.3686 ± $2.90x10^{-3}$	1.1774 ± $2.11x10^{-3}$
BG5	1.9968 ± $4.52x10^{-3}$	1.9975 ± $5.11x10^{-3}$	1.8925 ± $2.51x10^{-3}$	1.4782 ± $2.53x10^{-3}$	1.2455 ± $4.22x10^{-3}$	1.0258 ± $4.12x10^{-3}$
